# Exome sequencing and characterization of 49,960 individuals in the UK Biobank

**DOI:** 10.1038/s41586-020-2853-0

**Published:** 2020-10-21

**Authors:** Cristopher V. Van Hout, Ioanna Tachmazidou, Joshua D. Backman, Joshua D. Hoffman, Daren Liu, Ashutosh K. Pandey, Claudia Gonzaga-Jauregui, Shareef Khalid, Bin Ye, Nilanjana Banerjee, Alexander H. Li, Colm O’Dushlaine, Anthony Marcketta, Jeffrey Staples, Claudia Schurmann, Alicia Hawes, Evan Maxwell, Leland Barnard, Alexander Lopez, John Penn, Lukas Habegger, Andrew L. Blumenfeld, Xiaodong Bai, Sean O’Keeffe, Ashish Yadav, Kavita Praveen, Marcus Jones, William J. Salerno, Wendy K. Chung, Ida Surakka, Cristen J. Willer, Kristian Hveem, Joseph B. Leader, David J. Carey, David H. Ledbetter, Geisinger-Regeneron DiscovEHR Collaboration, Lon Cardon, George D. Yancopoulos, Aris Economides, Giovanni Coppola, Alan R. Shuldiner, Suganthi Balasubramanian, Michael Cantor, Regeneron Genetics Center, Matthew R. Nelson, John Whittaker, Jeffrey G. Reid, Jonathan Marchini, John D. Overton, Robert A. Scott, Gonçalo R. Abecasis, Laura Yerges-Armstrong, Aris Baras

**Affiliations:** 1 Regeneron Genetics Center, Tarrytown, NY USA; 2 GlaxoSmithKline, Stevenage, UK; 3 GlaxoSmithKline, Collegeville, PA USA; 4 Regeneron Pharmaceuticals, Tarrytown, NY USA; 5 Department of Pediatrics, Columbia University Irving Medical Center, New York, NY USA; 6 Department of Medicine, Columbia University Irving Medical Center, New York, NY USA; 7 University of Michigan, Ann Arbor, MI USA; 8 Norwegian University of Science and Technology, Trondheim, Norway; 9 Geisinger, Dannville, PA USA; 10 Present Address: AstraZeneca, Cambridge, UK; 11 Present Address: Foresite Labs, Cambridge, MA USA; 12 Present Address: Digital Health Center, Hasso Plattner Institute, University of Potsdam, Potsdam, Germany; 13 Present Address: Hasso Plattner Institute for Digital Health at Mount Sinai, Icahn School of Medicine at Mount Sinai, New York, NY USA; 14 Present Address: DNANexus, Mountain View, CA USA; 15 Present Address: Deerfield, New York, NY USA

**Keywords:** Genomics, Rare variants, Next-generation sequencing, Genetics research

## Abstract

The UK Biobank is a prospective study of 502,543 individuals, combining extensive phenotypic and genotypic data with streamlined access for researchers around the world^1^. Here we describe the release of exome-sequence data for the first 49,960 study participants, revealing approximately 4 million coding variants (of which around 98.6% have a frequency of less than 1%). The data include 198,269 autosomal predicted loss-of-function (LOF) variants, a more than 14-fold increase compared to the imputed sequence. Nearly all genes (more than 97%) had at least one carrier with a LOF variant, and most genes (more than 69%) had at least ten carriers with a LOF variant. We illustrate the power of characterizing LOF variants in this population through association analyses across 1,730 phenotypes. In addition to replicating established associations, we found novel LOF variants with large effects on disease traits, including *PIEZO1* on varicose veins, *COL6A1* on corneal resistance, *MEPE* on bone density, and *IQGAP2* and *GMPR* on blood cell traits. We further demonstrate the value of exome sequencing by surveying the prevalence of pathogenic variants of clinical importance, and show that 2% of this population has a medically actionable variant. Furthermore, we characterize the penetrance of cancer in carriers of pathogenic *BRCA1* and *BRCA2* variants. Exome sequences from the first 49,960 participants highlight the promise of genome sequencing in large population-based studies and are now accessible to the scientific community.

## Abstract (web summary)

Exome sequences from the first 49,960 participants in the UK Biobank highlight the promise of genome sequencing in large population-based studies and are now accessible to the scientific community.

## Main

The UK Biobank (UKB) is a prospective population-based study of more than 500,000 individuals with extensive and readily accessible phenotypic and genetic data^1^. The release of genome-wide genotyping array data^2^ for study participants has accelerated genomic discovery through association studies and enabled advances in population genetic analyses, the exploration of genetic overlap between traits and Mendelian randomization studies^3,4^. Although array data in combination with genotype imputation capture the spectrum of common genetic variants, rare variation that is more likely to modify protein sequences and have large phenotypic consequences is captured less well by these approaches.

Here, we extend the UKB resource with the first study of whole-exome sequencing (WES) for 49,960 UKB participants, generated by the Regeneron Genetics Center, as part of a collaboration with GlaxoSmithKline. These data are available to approved researchers through the UKB Data Showcase (http://ukbiobank.ac.uk/). WES enables the direct assessment of protein-altering variants, the functional consequences of which are more readily interpretable than non-coding variants, providing a clearer path towards mechanistic and therapeutic insights, as well as having a potential use in therapeutic target discovery and validation^5–8^ and in precision medicine^9,10^. Here, we provide an overview of sequence variation in UKB exomes, review predicted damaging variants and their consequences in the general population and perform comprehensive predicted LOF burden testing with 1,730 phenotypes, illustrating the use of this resource in studies of common and rare phenotypes with a focus on deleterious coding variation.

## Demographics and clinical characteristics

We selected 50,000 participants from the UKB resource and prioritized individuals with more complete phenotype data: those with whole-body magnetic resonance imaging (MRI) data from the UKB Imaging Study, enhanced baseline measurements, hospital episode statistics (HES) and linked primary healthcare records. Additionally, we selected one disease area for enrichment, including individuals with admission to hospital with a primary diagnosis of asthma (International Clinical Diagnosis 10 (ICD10) codes J45 or J46). This resulted in 8,250 participants with asthma, or approximately 16% of sequenced participants, compared with an incidence of 13% among all 502,543 UKB participants (Table 1). During data generation, samples from 40 participants were excluded due to failed quality-control measures or participant withdrawal, which resulted in a final set of 49,960 individuals of predominantly European ancestry. All study participants provided informed consent. The sequenced participants are representative of the overall 502,543 UKB participants (Table 1) for age, sex and ancestry. Owing to the ascertainment strategy, sequenced participants were more likely to have HES diagnosis codes (84.2% of participants in the WES study compared with 78.0% overall), were enriched for individuals with asthma and had numerous enhanced physical measures (eye measures, hearing tests and electrocardiogram data, Table 1). The exome-sequenced participants did not differ from all participants in the median number of primary and secondary ICD10 codes. The WES individuals include 194 parent–offspring pairs, including 26 mother–father–child trios, 613 full-sibling pairs, 1 monozygotic twin pair and 195 second-degree relatives based on identity-by-descent estimates^11^ (Supplementary Fig. 1).

**Table 1:** Clinical characteristics of WES and all UKB participants

Demographic and clinical characteristics	UKB 50,000 WES participants	UKB 500,000 participants
Number of participants	49,960	502,543
Number of women (%)	27,243 (54.5)	273,460 (54.4)
Age at assessment in years (Q1–Q3)^a^	58 (45–71)	58 (45–71)
Body-mass index in kg m^−2^ (Q1–Q3)^a^	26 (21–31)	26 (21–31)
Number of imaged participants (%)^a^	12,075 (24.1)^b^	21,407 (4.3)^b,c^
Number of current and past smokers (%)^a^	17,515 (35.0)	216,482 (43.1)
Townsend deprivation index (Q1–Q3)^a^	−2.0 (−6.1, −2.1)	−2.1 (−6.2, −1.9)
Inpatient (ICD10) 3-digit codes per patient (Q1–Q3)	5 (2–9)	5 (2–9)
Patients with ≥1 inpatient ICD10 diagnoses (%)	42,066 (84.2)	391,983 (78.0)
**Genetic ancestry assignment**^d^		
African (%)	1.49	1.24
East Asian (%)	0.54	0.51
European (%)	93.6	94.5
**Cardiometabolic phenotypes**		
Coronary disease (%)	3,340 (6.7)	35,879 (7.1)
Heart failure (%)	300 (0.6)	4,399 (0.8)
Type 2 diabetes (%)	1,541 (3.0)	17,261 (3.4)
**Respiratory**		
Asthma (%)	8,250 (16.5)	68,149 (13.5)
COPD (%)	741 (1.4)	7,438 (1.4)
**Oncology phenotypes**		
Breast cancer in women (% in women)	1,657 (6.1)	16,772 (6.1)
Ovarian cancer (% in women)	162 (0.6)	1,777 (0.6)
Pancreatic cancer (%)	602 (1.2)	4,611 (0.9)
Prostate cancer (% in men)	848 (3.7)	8,855 (3.9)
Melanoma (%)	598 (1.1)	5,715 (1.1)
**Enhanced measures**^a^		
Hearing test (%)	40,546 (81.1)	167,011 (33.2)
Visual acuity measured (%)	39,461 (78.9)	117,092 (23.2)
IOP measured (left) (%)	37,940 (75.9)	111,942 (22.2)
Autorefraction (%)	36,067 (72.1)	105,989 (21.0)
Retinal OCT (%)	32,748 (65.5)	67,708 (13.4)
Electrocardiogram at rest (%)	10,829 (27.1)	13,572 (2.1)

Demographics and clinical characteristics of UKB 50,000-sequenced participants and overall 500,000 participants. See Supplementary Methods for definition of UKB clinical phenotype definitions and Supplementary Table 1 for additional characteristics. Values are expressed as the median (first to third quantile (Q1–Q3)) or as counts (and percentages). COPD, chronic obstructive pulmonary disease; IOP, intra-ocular pressure; OCT, optical coherence tomography.

^a^Demographic and enhanced measure counts were based on data from the initial assessment visit.

^b^The number of samples with WES data and at least one non-missing image-derived phenotype value from data downloaded from the UKB in November 2018.

^c^The number of samples with at least one non-missing image-derived phenotype value from data downloaded from UKB in November 2018.

^d^The genetic ancestry assignment is based on the number of samples in 3 predefined regions of a plot of the first two genetic principal component scores, where the regions are selected to represent African, East Asian and European ancestry (Supplementary Fig. 2).

## Characterization of coding variation in WES data

Exomes were captured using a slightly modified version of the IDT xGen Exome Research Panel v.1.0. The basic design targets 39 megabases of the human genome (19,396 genes among autosomes and sex chromosomes) and was supplemented with additional probes to boost coverage at underperforming loci. In each sample and among targeted bases, coverage exceeds 20× at 94.6% of sites on average (s.d., 2.1%). We observe 4,752,777 variants within targeted regions (Table 2). They include 1,224,107 synonymous (97.8% with minor allele frequency (MAF) < 1%), 2,492,667 missense (98.9% with MAF < 1%) and 198,269 predicted LOF variants that affect at least one coding transcript (initiation codon loss, premature stop codons, splicing and frameshifting insertion or deletion (indel) variants; 99.6% with MAF < 1%); the increasing proportion of rare variants in the LOF and missense categories is consistent with purifying selection (Supplementary Fig. 3a). The median number of variants per individual includes 9,584 synonymous (interquartile range (IQR), 128), 8,702 missense (IQR, 136) and 120 LOF variants (IQR, 12) and is comparable to previous exome-sequencing studies^12,13^. Restricting analysis to LOF variants that affect all ENSEMBL v.85 transcripts for a gene, the number of LOF variants decreases to 132,336 overall and 95 per individual (a reduction of around 33.2% and about 20.8%, respectively), which is consistent with previous studies. Among LOF variants, we observed similar sequencing depth and allelic balance in comparison to non-LOF variants (Supplementary Table 2). Including non-targeted regions adjacent to exons, we observe 10,189,098 indel and single-nucleotide variants (SNVs) after quality control, 98.5% with a MAF < 1%. As expected, the X chromosome exhibited lower proportions of LOF-to-missense variants (2,492,667 missense and 198,269 LOF variants in autosomes, an approximately 12.6:1 ratio (Table 2); 59,796 missense and 3,670 LOF variants on the X chromosome, an around 16.3:1 ratio) (Supplementary Table 3). This phenomenon is the expected result of the increased efficiency of selection against deleterious variants on the X chromosome.

**Table 2:** Summary statistics for variants in sequenced exomes of 49,960 UKB participants

	Autosomal variants in WES		Median autosomal variants per participant (IQR)	
	Variants	Variants with MAF < 1%	Variants	Variants with MAF < 1%
Total	10,189,098	10,037,865	49,477 (739)	1,695 (176)
Targeted regions^a^	4,752,777	4,682,580	24,339 (285)	783 (63)
**Variant type**^a^				
SNVs	4,540,330	4,473,417	23,541 (276)	741 (61)
Indels	212,447	209,163	797 (29)	43 (10)
Multi-allelic	604,728	593,926	3,410 (64)	120 (18)
**Predicted function**				
Synonymous	1,224,107	1,197,941	9,584 (128)	227 (27)
Missense	2,492,667	2,466,331	8,702 (136)	379 (39)
LOF (any transcript)	198,269	197,574	175 (14)	20 (7)
LOF (all transcripts)	132,336	131,942	95 (11)	13 (5)
LOF (LOFTEE)	169,881	169,406	120 (12)	16 (7)

Counts of autosomal variants observed across all individuals by type or functional class for all and for variants with MAF < 1%. The number of targeted bases by the exome-capture design was *n* = 38,997,831. All variants passed the quality-control criteria (see Supplementary Methods), individual and variant missingness <10% and Hardy–Weinberg *P* > 10^−15^. The median count and IQR per individual for all variants and for variants with MAF < 1% are shown.

^a^Counts are restricted to WES-targeted regions.

## Enhancement of coding variation in WES data

To evaluate the enhancements of WES data to the UKB genetic variation resource, we compared the number of targeted autosomal coding variants observed in 49,909 individuals for whom WES, array genotypes and the imputed sequence were all available from the UKB^2^; therefore, the variant counts for this subset of data differ from Table 2. Variants in the WES dataset were included in this analysis if the data passed the quality-control criteria (see Supplementary Methods) and variants in the imputed sequence dataset were included if the data had an info score > 0.3. Among all autosomal variants, we observed increases in the total number of coding (3,913,039 compared with 647,230), synonymous (1,243,633 compared with 249,263), missense (2,491,290 compared with 384,406) and LOF (198,116 compared with 13,561) variants in the WES dataset compared with the imputed sequence data, respectively (Supplementary Table 4). This represents a 14.6-fold increase in the number of autosomal LOF variants identified by WES compared to those in the imputed sequence. In addition, there was a 16.1-fold increase in the ascertainment of indels in the WES dataset (212,447 (Table 2)) compared to the imputed sequence dataset (13,179) (Supplementary Table 5). Among nearly four-million coding variants observed in the WES data, only 13.0% were also observed in the imputed sequence dataset, highlighting the added value of WES for the ascertainment of rare coding variation.

## Concordance of WES, array and imputed genotypes

We summarized the concordance between the WES and array genotypes as well as the WES and imputed allele dosages for the 46,911 individuals of European ancestry for whom all three genetic resources were available, using the squared correlation (*R*^2^) of allele counts (see Supplementary Methods). This measure facilitates the interpretation of assessments of accuracy for both rare and common variation^14,15^. As expected, concordance measured by *R*^2^ between genomic measures declines with a decrease in MAF (Supplementary Fig. 4).

*R*^2^ concordance between the WES and imputed sequence data ranged from 32.2% for MAF < 0.01% to 95.2% for MAF > 1%, with an average of 53.1% across all allele frequencies. Furthermore, the WES dataset has much greater concordance with array genotypes, as both methods directly assay the variation rather than make a computational prediction. This is particularly true in the rarest allele frequencies for which the accuracy of imputation is limited when using current imputation reference panels. *R*^2^ concordance between WES and array genotyped variants was substantially higher, ranging from 73.2% for MAF < 0.01% to 98.7% for MAF > 1%, with an average of 92.3% across all allele frequencies.

## LOF variants in WES and imputed sequence data

LOF variants constitute a major class of genetic variation that is of great interest because of their disruption of gene function, their causal role in many Mendelian disorders and the success of leveraging protective LOF variants to identify new drug targets^5,6,16^. Rare LOF variation is best captured by direct sequencing approaches, such as WES, and we sought to quantify the improved yield of LOF variation from WES compared to array genotyping and the imputed sequence. We compared the number of LOF variants ascertained through WES and the imputed sequence among 46,911 UKB participants of European ancestry. Notably, not all individuals with WES data have data for the imputed sequence available—105 individuals for whom WES data were available failed the quality-control criteria for the genotyping chip and were not included in the UKB genotyping chip or imputed sequence release. We observed a larger number of LOF variants that affected any transcript in the WES dataset compared with the imputed sequence data (248,730 compared with 14,872, respectively) (Supplementary Table 6). Furthermore, we observed a greater number of genes with at least one carrier of a heterozygous LOF variant (17,718 genes from the WES dataset; 7,500 genes from the imputation dataset with an info score > 0.3) and genes with at least one carrier of a homozygous LOF variant (789 from WES; 612 from imputation) (Supplementary Table 7). At equivalent sample sizes (*n* = 46,911 individuals of European ancestry for whom both WES and imputed sequence data were available), the WES dataset included a greater number of genes with LOF variants, across all carrier count thresholds. Notably, the WES data from 46,911 individuals yielded more genes (17,718) with heterozygous LOF variants than the imputed sequence dataset (with info score > 0.3) for all 462,427 UKB participants of European ancestry (8,724 genes). Tracking the increase in the number of genes with carriers of heterozygous LOF variants with the increase in the number of sequenced samples suggests that we are approaching saturation for this metric, and have probably observed at least one carrier with a heterozygous LOF variant for most of the genes that tolerate these variants, and for most genes overall (Supplementary Fig. 3c). By contrast, the number of genes for which homozygous LOF variants are observed still appears to increase rapidly as more samples in the UKB are sequenced, which suggests that homozygous instances of LOF variants for many more genes can be identified by sequencing additional individuals (Supplementary Fig. 3d).

## Projection of variants in 500,000 UKB participants

**Fig. 1:**
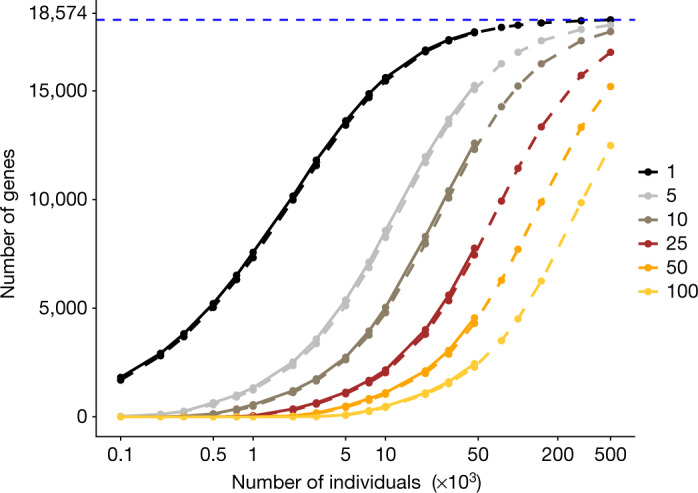
Predicted number of genes in carriers of heterozygous LOF variants in around 500,000 whole-exome sequences from existing WES data. The number of autosomal genes with at least 1, 5, 10, and so on, carriers of heterozygous LOF variants that passed Goldilocks quality control (see Supplementary Methods), had genotype missingness of <10% and Hardy–Weinberg equilibrium *P* > 10^−15^ increases with sample size. UKB participants of European ancestry with WES data (*n* = 46,911) were downsampled at random to the number of individuals specified on the *x* axis. The number of genes containing at least the indicated count of carriers of heterozygous LOF variants with MAF < 1% as indicated in the legend are plotted on the *y* axis. The number of autosomal genes is 18,574 in this gene model. The blue dashed line indicates the predicted number of genes (18,273) with at least 1 carrier of a heterozygous LOF variant in 500,000 exomes. Solid curves connect the observed number of genes; dashed curves connect predicted counts from a β-binomial mixture model (see Supplementary Methods).

By extrapolating the data, we can estimate the number of genes for which multiple carriers of LOF variants will be observed once our experiment is complete and all approximately 500,000 participants are sequenced. Cautiously, we currently predict that more than 17,000, 15,000 and 12,000 genes will have at least 10, 50 and 100 carriers of heterozygous LOF variants in the full dataset, respectively (Fig. 1, Supplementary Methods).

## Comparison to other large-scale WES resources

Our WES results are consistent with those of a recent large-scale survey^17^ of genetic variation in 141,456 individuals from the Genome Aggregation Database (gnomAD). When we annotate both exome variant lists with the same annotation pipelines and subset the results to similar numbers of individuals and ancestry, we observe 17,718 genes with LOF variants in any transcript (LOF_RGC_) in 46,911 individuals of European ancestry in the UKB with WES data compared with 17,946 genes with LOF variants in any transcript in 56,885 individuals of non-Finnish European ancestry in gnomAD exomes. Further restricting the data to high-confidence LOF variants with LOFTEE (LOF_LOFTEE_) (see Supplementary Methods), we obtain 17,323 genes with LOF variants in the UKB and 17,620 in gnomAD (see Supplementary Methods). Consistent with expectation, we observe around 10% more genes with at least 10 LOF variants, including single-nucleotide polymorphisms and indels, and around 12% more genes with at least 10 individuals carrying LOF variants using the LOF_RGC_ definition compared to LOF_LOFTEE_, respectively (Supplementary Fig. 5).

## Survey of medically actionable pathogenic variants

To date, more than 5,000 genetic disorders have known molecular causes and associated genes. The American College of Medical Genetics (ACMG) has proposed 59 genes (ACMG SF2.0)^18,19^ (hereafter ACMG59) that are associated with highly penetrant disease phenotypes and for which available treatments and/or prevention guidelines can reduce the morbidity and mortality in genetically susceptible individuals. Large-scale human genomic sequencing efforts coupled with electronic health record data provide opportunities to assess penetrance and prevalence of pathogenic and likely pathogenic variants in known monogenic disease genes, as well as investigate the phenotypic effects of variants of unknown significance. In addition, phenotypically agnostic population sampling provides opportunities to better characterize the phenotypic spectrum of these disorders and estimate the associated disease penetrance in the population. Furthermore, these efforts enable the implementation of precision medicine by identifying individuals who carry medically actionable pathogenic variants and providing medical care and surveillance pre-emptively. The informed consent for the UKB does not allow the return of these results to individuals or their healthcare providers as it does in the Geisinger-Regeneron DiscovEHR project^10^.

We interrogated variant data for the 49,960 individuals with WES from the UKB to identify a ‘strict’ set of reported pathogenic missense and LOF variants (that is, those with at least two stars in ClinVar and no conflicting interpretations) as well as a set of likely pathogenic LOF variants (that is, those in genes for which truncating variants are known to cause disease) according to current ACMG guidelines^19^ (see Supplementary Methods). We identified 548 such variants (315 reported pathogenic, 233 likely pathogenic) in 992 carriers from the sample of 49,960 individuals (Supplementary Table 8). Nine individuals carried variants in two genes. Of note, 37 of the likely pathogenic variants would qualify as previously reported pathogenic variants using a broader definition (Supplementary Table 9). Pathogenic or likely pathogenic variants were observed in 47 out of 59 genes listed in ACMG59, with a median number of 4 variants per gene and a median of 7 carriers per gene (these calculations included the 12 genes for which there were no observed carriers). Overall, 2.0% of the sequenced individuals carry a reportable pathogenic or likely pathogenic variant in at least one gene from the 59 gene set of ACMG59. Using the same methodology for data from 91,514 participants from the DiscovEHR study^20^ that have been sequenced to date, we observed a slightly higher percentage of individuals, 2.76%, who carry a potentially actionable rare pathogenic or likely pathogenic variant in the 59 gene set of ACMG59 (Supplementary Table 10). This difference may reflect differences between a study of individuals who seek clinical care (DiscovEHR) and a population-based study not ascertained in the context of active clinical care (UKB).

Among the 47 reportable genes from the 59 gene set of ACMG59, variants in cancer-associated genes were the most prevalent in UKB with WES; *BRCA2* (93 variants, 166 carriers), *BRCA1* (39 variants, 59 carriers), *PMS2* (21 variants, 59 carriers) and *MSH6* (35 variants, 52 carriers); and variants in *LDLR*, which is associated with familial hypercholesterolaemia (Mendelian inheritance in man (MIM) code 143890), were the second most prevalent (35 variants, 68 carriers). Cardiac dysfunction disorders were also highly represented mainly by variants in *KCNQ1*, which causes long QT syndrome (MIM192500) (25 variants, 55 carriers), *PKP2*, which causes arrhythmogenic right ventricular dysplasia (MIM 609040) (21 variants, 54 carriers), and *MYBPC3*, which causes cardiomyopathy (MIM 115197) (25 variants, 50 carriers) (Supplementary Fig. 6a). Of the 992 carriers of medically actionable pathogenic variants, 985 were heterozygous for pathogenic variants in genes that are responsible for dominant conditions. Additionally, we identified six individuals who were homozygous for pathogenic variants in genes associated with autosomal recessive disorders (familial adenomatous polyposis 2, MIM 608456) and mismatch repair cancer syndrome (MIM 276300), which are caused by to biallelic pathogenic variants in *MUTYH* and *PMS2*, respectively) and one individual who was hemizygous for an X-linked condition (Fabry disease (MIM 301500), which is caused by pathogenic variants in *GLA*). Indeed, the male individual who was hemizygous for the pathogenic variant (c.335G>A; p.Arg112His) in *GLA* has diagnoses of angina pectoris, atrial fibrillation, chest pain and chronic ischaemic heart disease. Similarly, one individual who was homozygous for a pathogenic missense variant (c.1145G>A; p.Gly382Asp) in *MUTYH* has a history of benign neoplasm of the colon, diverticular disease of the intestine, colonic polyps and intestinal obstruction. These examples illustrate how the extensive health data available for UKB participants provide a valuable resource to assess variant pathogenicity and disease risk at the population level, and the potential to model outcomes for individuals who have pathogenic variants.

We evaluated the penetrance of pathogenic and likely pathogenic variants in *BRCA1* and *BRCA2* (Supplementary Fig. 6b and Supplementary Table 11) to compare five *BRCA1* or *BRCA2* associated cancers^21^ as well as to explore whether risk was conferred for other cancers. Although *BRCA1* and *BRCA2* have differences in risk among cancer subtypes, due to low sample sizes, we analysed summed counts in 114 male and 110 female participants with reported pathogenic and likely pathogenic variants in *BRCA1* or *BRCA2*. We found that the prevalence of cancers is increased in carriers of pathogenic or likely pathogenic variants in *BRCA1* or *BRCA2* for five cancers (breast cancer in women, ovarian cancer, prostate cancer, melanoma and pancreatic cancer derived from self-report or HES data) that have previously been associated with mutations in *BRCA1* or *BRCA2*. The cancer prevalence for any of the five cancers was 21.1% in carriers compared with 6.6% in non-carriers; odds ratio (OR) = 3.77 (95% confidence interval (CI) = 2.73–5.21), *P* = 1.7 × 10^−12^ (3,337 cases, 46,599 controls)). There was no significant difference between carriers and non-carriers when all other cancers, excluding these five, were combined (15.8% in carriers compared with 17.7% in non-carriers, OR = 0.87 (95% CI = 0.58–1.30), *P* = 0.55 (8,300 cases, 38,424 controls)). The most-prevalent cancer types in this group were unspecified malignant neoplasms of skin (C443 and C449), and carcinoma in situ of the cervix (D069). Increased risk was observed in the carriers of *BRCA1* or *BRCA2* variants for each of the five previously associated cancers analysed individually (ovarian cancer in women, OR = 10.07 (95% CI = 4.36–23.29), *P* = 4.9 × 10^−5^ (162 cases, 27,076 controls); breast cancer in women, OR = 4.42 (95% CI = 2.80–6.97), *P* = 2.9 × 10^−8^ (1,654 cases, 25,584 controls); prostate cancer in men, OR = 3.48 (95% CI = 1.98–6.11), *P* = 1.5 × 10^−4^ (888 cases, 21,818 controls); melanoma, OR = 1.9 (95% CI = 0.78–4.62), *P* = 0.20 (599 cases, 49,345 controls); and pancreatic cancer, OR = 3.48 (95% CI = 1.78–6.82), *P* = 1.7 × 10^−3^ (602 cases, 49,342 controls) (*P* values were calculated using Fisher’s exact tests). These differences in overall cancer risk also manifest as clearly earlier disease onset and rates of cancer-free survival. Comparing the cumulative proportion of female participants who were free of breast and ovarian cancer, we estimate a hazard ratio of 4.31 in carriers of *BRCA1* or *BRCA2* variants to non-carriers (95% CI = 2.97–6.26, *P* = 1.4 × 10^−16^) (Supplementary Fig. 6c). Comparing the cumulative proportion of male participants who were free of prostate cancer, the hazard ratio was 3.68 in carriers of *BRCA1* or *BRCA2* variants to non-carriers (95% CI = 2.17–6.24, *P* < 3.3 × 10^−7^) (Supplementary Fig. 6d). With the UKB WES resource, cancer risk can be more-deeply explored across broader sets of variants as well as with larger WES datasets and continued accrual of incident cancers. Our results corroborate those of another recent population-based application of WES linked to health records^10^ to evaluate cancer risk in individuals with pathogenic variants in *BRCA1* or *BRCA2*, demonstrating the value of WES to identify high-penetrance rare alleles that are associated with clinical phenotypes; such efforts can be applied across other genetic disorders, enabling the implementation of precision medicine at the population level. We also note that penetrance estimates from population-based studies such as ours appear to be lower than those from previous studies, which may have relied on the screening of high-risk patients or families (for example, we estimate an around 20% risk of breast cancer in carriers of medically actionable variants in *BRCA1* or *BRCA2* in our sample (median age, 58 years), compared with previous meta-analytic estimates of 30–40% risk at an age of 60 years)^22^.

## Phenotypic associations with LOF variation

The combination of WES, which enables the comprehensive capture of LOF variants, with rich health information allows for the broad investigation of the phenotypic consequences of human genetic variation. We conducted burden tests for rare (MAF < 1%) LOF variants in 16,219 autosomal genes with at least 4 carriers of LOF variants and in 1,730 traits (1,059 discrete traits with at least 50 cases defined by HES and self-reported data; 671 quantitative, anthropometric and blood traits) in 46,876 individuals of European ancestry. For each gene–trait association, we also evaluated the signal for the single variants included in the burden test. We identified 26 unique gene burden–trait associations with *P* < 10^−7^; among these, 24 were more significant than any single LOF variant included in the burden test. Given the number of genes and traits analysed, we expected around 2.5 false-positive results to reach *P* < 10^−7^ (corresponding to a false-discovery rate of approximately 10%). Reassuringly, the results include several well-established associations (Supplementary Tables 12, 13), in addition to new signals (Table 3), for which we sought replication (Supplementary Tables 14, 15).

**Table 3:** Novel LOF gene burden associations

Gene	ICD10 code (binary phenotype)	RR|RA|AA	OR (95% CI)	WES burden *P*	SNV (*n*)	Lowest *P* SNV	Imputed 50,000 burden *P*
*PIEZO1*	I83.9 (asymptomatic varicose veins of lower extremities)	Control: 43,189|142|0 Case: 1,266|20|0	4.9 (2.8–8.6)	3.2 × 10^−8^	73	2.6 × 10^−3^	0.083
*FAM160B1*	T81.3 (disruption of wound)	Control: 44,723|8|0 Case: 65|3|0	280 (35–2,200)	9.4 × 10^−8^	9	NA^a^	NA^b^
Gene	Quantitative phenotype	RR|RA|AA	*β* (95% CI)	WES burden *P*	SNV (*n*)	Lowest *P* SNV	Imputed 50,000 burden *P*
*MEPE*	Heel-bone mineral density *t*-score	41,775|150|0	−0.46 (−0.62, −0.3)	1.2 × 10^−8^	26	3.6 × 10^−5^	3.4 × 10^−3^
*COL6A1*	Mean corneal resistance factor	35,533|17|0	−1.5 (−2, −1)	4.7 × 10^−10^	20	1.8 × 10^−4^	NA^b^
*COL6A1*	Mean corneal hysteresis	35,532|17|0	−1.3 (−1.8, −0.86)	2.9 × 10^−8^	20	5.3 × 10^−4^	NA^b^
*IQGAP2*	Mean platelet thrombocyte volume	45,362|164|0	0.72 (0.57–0.86)	9.2 × 10^−22^	57	6.6 × 10^−9^	0.11
*GMPR*	Mean corpuscular haemoglobin	45,162|183|0	0.4 (0.25–0.54)	3.8 × 10^−8^	13	2.1 × 10^−7^	2.6 × 10^−5^

LOF gene burden association with available clinical and continuous traits in 46,979 UKB participants of European ancestry with WES.

^a^No SNV reached the threshold for association analysis (minor allele count of at least four).

^b^Not applicable (NA), no LOF variants were observed in the imputed sequence.

For example, we observe that carriers of *MLH1* LOF variants, which are associated with Muir–Torre and Lynch syndromes^23^ (MIM 158320 and 609310, respectively), were at increased risk of malignant neoplasms of the digestive organs (OR = 70, *P* = 8.2 × 10^−11^). Carriers of *PKD1* LOF variants, the major cause of autosomal dominant polycystic kidney disease^24^ (MIM 173900), were at increased risk of chronic kidney disease (OR = 86, *P* = 6.4 × 10^−10^). Carriers of *TTN* LOF variants are at increased risk of cardiomyopathy (OR = 12, *P* = 6.7 × 10^−9^), which is consistent with previous reports^25^. In addition to Mendelian disorders, other findings with strong support in the literature include *HBB* with red-blood-cell phenotypes^26^, *IL33* with eosinophils (driven by rs146597587)^27^, *KALRN* with platelet volume (driven by rs56407180)^28^, *GP1BA* with platelet volume^29^, *TUBB1* with multiple platelet phenotypes^30^ and *COL4A4* with urine microalbumin^31^. In some cases, we see patterns of association with traits that may be secondary to known phenotypic associations. For example, *ASXL1* and *CHEK2* are genes involved in myeloproliferative disorders^32^ and cancer^33^, respectively, which may explain the observed associations with haematological traits (which may be secondary to myelodysplastic disease or chemotherapy). Indeed, carriers of LOF variants in *ASXL1*, a known driver of clonal haematopoiesis of indeterminant potential, are 5 years older on average than non-carriers. This is consistent with the possibility that many of the observed *ASXL1* LOF variants are somatic mutations. Of the 103 carriers of *ASXL1* LOF variants, 45 (44%) were identified as carriers of LOF variants for clonal haematopoiesis of indeterminant potential (see Supplementary Methods). We observed no significant differences in age for carriers of LOF variants in the other genes included in Tables 3 or Supplementary Tables 12, 13 (Supplementary Table 16).

Many other known phenotypic associations are supported by the data at more-modest significance thresholds (Supplementary Table 13). These include, for example, associations between LOF variants in *LDLR* with coronary artery disease^34^, *GP1BB* with platelet count^35^, *KRT5* with fibrosis and scarring of skin^36^, as well as *PALB2* and *BRCA2* with cancer risk^37,38^. We observed no exome-wide significant LOF burden associations on chromosome X.

### LOF associations and novel gene discovery

Our LOF gene burden association analysis identified six gene–trait LOF associations with *P* < 10^−7^ that have not previously been reported (Table 3). We identified a novel association between *PIEZO1* LOF variants (cumulative allele frequency = 0.18%) and increased risk for varicose veins (OR = 4.9, *P* = 3.2 × 10^−8^). This finding is driven by a burden of rare LOF variants, with the most-significant *PIEZO1* single-variant LOF association achieving a *P* value of 2.6 × 10^−3^. ‘Leave-one-out’ sensitivity analyses indicated that no single variant accounted for the entire signal and step-wise regression analyses indicated that three separate variants (one of which had a minor allele count of greater than one) were contributing to the burden signal (Supplementary Table 17). We replicated this finding for varicose veins (1,572 cases, 75,704 controls) in WES data from the DiscovEHR study (OR = 3.8, *P* = 1.5 × 10^−6^) (Supplementary Table 14). This region had previously been implicated in varicose veins by common non-coding variants with small effects^39^ (rs2911463, OR = 0.996). *PIEZO1* encodes a 36-transmembrane-domain cation channel that is highly expressed in the endothelium and has a critical role in the development and adult physiology of the vascular system as it translates shear stress into electrical signals^40^. This previous report of the rs2911463 variant mapped the association to *PIEZO1* through evidence of gene function and analysis using DEPICT, but did not find strong evidence in expression quantitative trait loci that would clarify the mechanism in order to modulate the target therapeutically^39^. Rare missense variants have been reported in families segregating autosomal-dominant dehydrated hereditary stomatocytosis (MIM 194380), which is characterized by haemolytic anaemia with primary erythrocyte dehydration due to decreased osmotic fragility. Additionally, biallelic LOF variants in *PIEZO1* have been reported in families with lymphatic malformation syndrome (MIM 616843), a rare autosomal recessive disorder characterized by generalized lymphoedema, intestinal and/or pulmonary lymphangiectasia and pleural or pericardial effusions. Notably, some of the reported patients with lymphoedema presented with varicose veins and deep-vein thrombosis^41^. Our data not only provide support for *PIEZO1* as the causal gene in this locus, but also clarify the direction of effect, as the loss of gene function in carriers with heterozygous LOF variants leads to an increased risk of developing varicose veins.

We also identified a novel LOF burden association with *MEPE* (cumulative MAF = 0.18%) and decreased bone mineral density (BMD) (as measured by heel BMD *t*-score, −0.46 s.d., *P* = 1.2 × 10^−8^). Leave-one-out analyses suggested that the aggregate signal is driven by multiple variants (Supplementary Table 18), only one of which could be imputed (rs753138805, which encodes a frameshift that results in an early truncation of the protein). In analysis of all 500,000 UKB participants, rs753138805 was significantly associated with decreased BMD (−0.4 s.d., *P* = 2.0 × 10^−18^) and showed a trend for increased risk of osteoporosis (OR = 1.9, *P* = 0.10, *n* = 3,495 cases, Supplementary Table 15). These findings are corroborated by analysis of the HUNT study^42^, in which rs753138805 was associated with decreased BMD (−0.5 s.d., *P* = 2.1 × 10^−18^) and increased risk of any fracture (OR = 1.4, *P* = 1.6 × 10^−5^, *n* = 24,155 cases) (Supplementary Table 15). In the DiscovEHR study, we observed a directionally consistent, but nonsignificant, association for *MEPE* LOF variants with femoral-neck BMD *t*-score (−0.19, *P* = 0.22) (Supplementary Table 14). The *MEPE* locus was previously reported^43^ to have six independent signals with modest effects on BMD.

Although two of the previously reported non-coding variants are in moderate (*r*^2^ = 0.5) or high (*r*^2^ = 0.78) linkage disequilibrium with two variants that contribute to the burden test, the burden association is only partially attenuated in conditional analyses (*P* = 2.9 × 10^−4^, including all six previously reported variants). *MEPE* encodes a secreted calcium-binding phosphoprotein that has a key role in osteocyte differentiation and bone homeostasis^44,45^. Studies in *Mepe*^*−/−*^ knockout mice (*Mepe*^*tm1Tbrw*^) have yielded inconsistent results, with two groups reporting an increase in BMD^45,46^ and another reporting no change^47^.

Another new signal comprises LOF variants in *COL6A1* (cumulative allele frequency = 0.03%) that are associated with a 2.7 mm Hg decrease in corneal resistance factor (−1.5 s.d., *P* = 4.7 × 10^−10^) and corneal hysteresis (−1.3 s.d., *P* = 2.9 × 10^−8^), which are measures of corneal biomechanics^48^. *COL6A1* encodes a component of the collagen type-VI microfibrils, which have important roles in maintaining the structure and function of the extracellular matrix and which are major components of the human cornea^49^. This locus has previously been implicated in ocular traits; rs73157695 and nearby common variants have been associated with myopia^50^ (OR = 0.94; *P* ≈ 10^−13^) and intraocular pressure^51^. Leave-one-out analyses indicate that multiple variants are driving the association (Supplementary Table 19) and that these are not in strong linkage disequilibrium with previously reported variants (Supplementary Tables 20, 21). Protein levels of COL6A1 were reduced in eyes from patients with keratoconus^52^, and individuals with keratoconus and other corneal diseases, such as Fuchs’ corneal dystrophy, have reduced corneal hysteresis and corneal resistance factor^53^. Measures of corneal hysteresis and corneal resistance factor were not available in the DiscovEHR study for replication analyses.

The new LOF burden associations for *IQGAP2*, *GMPR* (driven by rs147049568) and *FAM160B1* relate to platelet morphology, haemoglobin levels and wound healing, respectively. Previous genome-wide association and exome-chip studies have implicated *IQGAP2* and *GMPR* in haematological variability^28,54^. Our results for these two genes, each of which could be replicated in the DiscovEHR study (Supplementary Table 14), provide additional evidence of causal roles for these genes and establishes the direction of effect with respect to gene function on haematological traits. We also observe nominal evidence for replication of *FAM160B1* LOF variants with disruption of wound healing in the DiscovEHR study (OR = 13.2, *P* = 0.02) and find that no single variant drives the association signal in leave-one-out analyses (Supplementary Table 22). However, little is known about the function of FAM160B1, and further characterization is necessary.

In equivalent sample sizes, the LOF burden results from the imputed sequence dataset would not have uncovered the LOF associations at *P* < 10^−7^ as identified by WES (Supplementary Table 23). Furthermore, for 19 out of 26 LOF burden results described herein, gene burden results from WES in the 50,000 participant subset were more significant than burden results from the imputed sequence dataset of all 500,000 UKB participants (Supplementary Table 23), demonstrating the value of rare variants captured by WES to power these associations. Notably, we observed qualitatively similar significance for burden tests using LOF_RGC_ and LOF_LOFTEE_ criteria (Supplementary Fig. 7).

## Discussion

Integration of large-scale genomic and precision medicine initiatives offer the potential to revolutionize medicine and healthcare. Such initiatives provide a foundation of knowledge that link genomic and molecular data to health-related data at the population scale, enabling researchers to more-completely and systematically study genetic variation and its functional consequences on health and disease. Here, we describe the initial study of large-scale WES data for 49,960 UKB participants, which—to our knowledge—is currently the largest open-access resource of WES data linked to health records and extensive longitudinal study measures. These data greatly extend the current genetic resource, particularly in ascertainment of rare coding variation, which we demonstrate is useful for resolving variant-to-gene links and the directionality of gene-to-phenotype associations.

After quality control, we observed nearly four million SNVs and indel coding variants. Only approximately 10.1% of the coding variants identified by WES were observed in the sequence imputed from the array of 46,911 participants with both WES and imputed sequence, highlighting the added value of WES. This enrichment was even more pronounced with LOF variation for which WES identified more than 240,000 targeted and untargeted LOF variants of which only approximately 4% of these were present in the imputed sequence dataset. Furthermore, 25.9% of the coding variants in the imputed sequence were not observed in the WES data that may represent a large proportion of rare variants that have poor imputation accuracy, as observed in our concordance and visual validation analyses. A small proportion of these variants, seen only in the imputed sequence, also represent variants that are not in the regions targeted by the exome capture design and sequencing, a limitation of the targeted-capture approach. Increasing the numbers of individuals and ancestral diversity in imputation reference panels is expected to improve imputation accuracy for rare variants.

As with previous studies of this size^20^, we observed a large number of LOF variants, including at least one carrier of a rare heterozygous LOF variant in 97% of autosomal genes (compared to more than 41% of autosomal genes in the imputed sequence data for the same participants). It is important to note that our LOF annotation strategy is geared towards increasing sensitivity for the identification of LOF variants and downstream association discovery. While the number of genes with heterozygous instances of LOF variants is approaching saturation at this sample size, WES in the entirety of the UKB resource will considerably increase the number of carriers of LOF variants and the ability to detect phenotypic associations. We also observe 789 autosomal genes with homozygous instances of rare LOF variants, and this number of genes will also increase with continued sequencing of all UKB participants; however, studies in populations with a high degree of parental relatedness^55,56^ will provide even more genes with homozygous LOF variants and complement efforts such as the UKB. LOF variation is an extremely important class of variation for identifying drivers of high genetic risk of disease, new disease genes and therapeutic targets. Very large sample sizes are needed to detect new LOF associations given their collective rare allele frequencies. The WES data provide a substantial enhancement to the number of LOF variants identified and the power to detect new associations, which will only improve with continued sequencing of all UKB participants.

We illustrate the unique value of this expanded WES resource in the UKB to assess pathogenic and likely pathogenic variants in a disease-agnostic large-scale population-based study with longitudinal follow-up. We conducted a survey of pathogenic and likely pathogenic variants in medically actionable ACMG59 genes. Using stringent variant-filtering criteria, we arrived at an estimated prevalence of 2% of individuals in this study population having a clinically actionable finding. This resource enables us to characterize disease-risk profiles for individuals who carry pathogenic and likely pathogenic variants in medically relevant disease genes, including cancer susceptibility genes, such as *BRCA1* and *BRCA2*. We observed that carriers of pathogenic or likely pathogenic variants had 3.77-fold greater odds of any of the five cancers previously associated with *BRCA1* or *BRCA2*; penetrance for any of the five cancers was 21.1% in carriers compared with 6.6% in non-carriers. We further explored whether these variants conferred risk to any other cancers and did not observe any such associations. This resource will be valuable for the assessment of variant pathogenicity, particularly for variants of unknown significance and novel variants, and in exploring the full spectrum of disease risk and phenotypic expression. One limitation of the resource for such purposes is limited ancestral diversity. This and other similar studies also highlight the value and potential to apply large-scale sequencing at the population scale to identify a meaningful proportion of individuals who are at high risk of diseases for which effective interventions are available that can reduce the morbidity and mortality of genetically susceptible individuals; such precision medicine approaches could substantially reduce the burden of many diseases.

We conducted gene-burden association testing for LOF variants across all genes and included more than 1,700 binary and quantitative traits. In addition to replication of numerous positive controls, we also highlight previously undescribed significant LOF associations that provide new biological and genetic insights of large effect on disease traits of interest, including *PIEZO1* for varicose veins, *MEPE* for BMD and *COL6A1* for corneal thickness. We identified a previously undescribed gene-burden association in *PIEZO1*, a mechanosensing ion channel that is present in endothelial cells in vascular walls; LOF variants confer a nearly fivefold increased odds of varicose veins in carriers of heterozygous LOF variants in *PIEZO1*. We also identified a previously undescribed LOF burden association in *MEPE*, which is associated with decreased BMD and an approximately 2-fold increased odds of osteoporosis and 1.5-fold increased risk of fractures. Overall, through WES and gene-burden tests of association for LOF variants, we identified 26 unique gene–trait associations that exceeded a *P* < 10^−7^, of which 24 were more significant than any single LOF variant included in the burden test, highlighting the value of WES and the ability to detect new associations that are driven by rare coding variation. Although several of these regions had previously been identified in genome-wide association studies of more than 10× the sample size, a key strength of the current approach is the identification of likely causal genes and the direction of effect: two key pieces of information required for translation towards new therapeutics. This survey of rare LOF associations was limited by sample size for most binary traits but was well-powered for many quantitative traits. Although surveys of LOF variation in the entire UKB study using array and imputed sequence data have identified LOF associations in previous reports^57,58^, the WES data identified new associations that are unique to the exome sequence and detected in only approximately one tenth of the sample size; our results highlight the considerable power of WES for the discovery of LOF variants and rare variant associations and the further promise of new biological insights through the sequencing of all participants in the UKB resource.

Efforts are underway to sequence the exomes of all 500,000 UKB participants; these efforts will greatly expand the rare coding variation ascertained, including the number of carriers of heterozygous LOF variants that can now be observed in nearly all genes and the number of genes for which naturally occurring homozygous knockouts can be observed. Coupled with the rich laboratory, biomarker, health-record, imaging and other health-related data that are continually added to the UKB resource, WES will enhance the power for discovery and will continue to yield many important findings and insights. The WES data are available to approved researchers through similar access protocols as existing UKB data (http://ukbiobank.ac.uk/).

### Reporting summary

Further information on research design is available in the Nature Research Reporting Summary linked to this paper.

## Online content

Any methods, additional references, Nature Research reporting summaries, source data, extended data, supplementary information, acknowledgements, peer review information; details of author contributions and competing interests; and statements of data and code availability are available at 10.1038/s41586-020-2853-0.

## Supplementary information

Supplementary Information This file contains exome sequencing methodology, alignment, variant calling, annotation, and visual validation pipelines. Methods for extrapolation of the number of genes with loss of function variants, Mendelian variant characterization, and gene burden and phenotype characterization as well as association and replication analyses are also included. It also includes Supplementary Tables 1-16, 23, 25, 26, 29 and 31 and Supplementary Figures 1-8.

Reporting Summary

Supplementary Table 17 Single point and leave one out association results for the PIEZO1 burden result.

Supplementary Table 18 Leave one out and single variant association results, linkage disequilibrium analysis all burden and associated single variants in the MEPE burden result.

Supplementary Table 19 Leave one out analyses for COL6A1 burden results with corneal resistance.

Supplementary Table 20 Conditional analyses for COL6A1 burden results with corneal resistance.

Supplementary Table 21 Linkage disequilibrium analyses for COL6A1 burden results with corneal resistance.

Supplementary Table 22 Leave one out association results for the FAM160B1 burden result.

Supplementary Table 24 Quantitative traits tested in UK Biobank burden analyses.

Supplementary Table 27 Results of visual validation of singleton putative loss of function variants.

Supplementary Table 28 ACMG variants passing visual validation observed in UKB 50k exomes.

Supplementary Table 30 Single variant analyses for those with minor allele count >5 for results in Table 3 and Supplementary Table 12.

## Data Availability

All WES, genotyping chip and imputed sequence data described in this paper are publicly available to registered researchers through the UKB data-access protocol. Additional information about registration for access to the data are available at http://www.ukbiobank.ac.uk/register-apply/. Further information about the WES data is available at http://www.ukbiobank.ac.uk/wp-content/uploads/2019/03/Access_064-UK-Biobank-50k-Exome-Release-FAQ-v3.pdf. Detailed information about the chip and imputed sequence data is available at http://www.ukbiobank.ac.uk/wp-content/uploads/2018/03/UKB-Genotyping-and-Imputation-Data-Release-FAQ-v3-2-1.pdf.
