# Supplementary material for: Exome sequencing and characterization of 49,960 individuals in the UK Biobank
Source: Nature. 2020 Oct 21;586(7831):749–56. doi: 10.1038/s41586-020-2853-0 (PMC7759458; doi:10.1038/s41586-020-2853-0)
Supplement: Supplementary file 1 — This file contains exome sequencing methodology, alignment, variant calling, annotation, and visual validation pipelines. Methods for extrapolation of the number of genes with loss of function variants, Mendelian variant characterization, and gene burden and phenotype characterization as well as association and replication analyses are also included. It also includes Supplementary Tables 1-16, 23, 25, 26, 29 and 31 and Supplementary Figures 1-8. [file 41586_2020_2853_MOESM1_ESM.pdf]

natureresearch
https://doi.org/10.1038/s41586-020-2853-0

Supplementary information

Exome sequencing and characterization of 49,960 individuals in the UK Biobank

In the format provided by the authors and unedited

Exome sequencing and characterization of 49,960 individuals in UK Biobank

Supplementary Information

Authors: Cristopher V. Van Hout[1], Ioanna Tachmazidou[2,9], Joshua D. Backman[1], Joshua D. Hoffman[3,10], Daren Liu[1], Ashutosh K. Pandey[3], Claudia Gonzaga-Jauregui[1], Shareef Khalid[1], Bin Ye[1], Nilanjana Banerjee[1], Alexander H. Li[1], Colm O'Dushlaine[1], Anthony Marcketta[1], Jeffrey Staples[1], Claudia Schurmann[1,11], Alicia Hawes[1], Evan Maxwell[1], Leland Barnard[1], Alexander Lopez[1], John Penn[1,12], Lukas Habegger[1], Andrew L. Blumenfeld[1], Xiaodong Bai[1], Sean O'Keeffe[1], Ashish Yadav[1], Kavita Praveen[1], Marcus Jones[4], William J. Salerno[1], Wendy K. Chung[5], Ida Surakka[6], Cristen J. Willer[6], Kristian Hveem[7], Joseph B. Leader[8], David J. Carey[8], David H. Ledbetter[8], Geisinger-Regeneron DiscovEHR Collaboration[*], Lon Cardon[2], George D. Yancopoulos[4], Aris Economides[4], Giovanni Coppola[1], Alan R. Shuldiner[1], Suganthi Balasubramanian[1], Michael Cantor[1], Regeneron Genetics Center[*], Matthew R. Nelson[3,13,†], John Whittaker[2,†], Jeffrey G. Reid[1,†], Jonathan Marchini[1,†], John D. Overton[1,†], Robert A. Scott[2,†], Gonçalo R. Abecasis[1,†], Laura Yerges-Armstrong[3,†], Aris Baras[1,†]

* Banner details appear in the Supplementary Information.

† These authors jointly supervised this work.

Affiliations:

1. Regeneron Genetics Center, Tarrytown, NY, USA

2. GlaxoSmithKline, Stevenage, UK

3. GlaxoSmithKline, Collegeville, PA, USA

4. Regeneron Pharmaceuticals, Tarrytown NY, USA

5. Departments of Pediatrics and Medicine, Columbia University Irving Medical Center, New York, New York, USA

6. University of Michigan, Ann Arbor, MI, USA

7. Norwegian University of Science and Technology, Trondheim, Norway

8. Geisinger, Dannville, PA, USA

9. Present address: AstraZenica, Cambridgeshire, UK

10. Present address: Foresite Labs, Cambridge, MA USA

11. Present address: Digital Health Center, Hasso Plattner Institute, University of Potsdam, Potsdam, Germany. Hasso Plattner Institute for Digital Health at Mount Sinai, Icahn School of Medicine at Mount Sinai, New York, NY, USA.

12. Present address: DNANexus, Mountain View, CA, USA

13. Present address: Deerfield, New York, NY, USA

METHODS

Table of Contents

Institutional review and oversight 3
Informed consent 4
WES sample preparation and sequencing 4
Sequence alignment, variant identification, and genotype assignment 5
Phenotype definition 6
Extrapolation of genes with LOF variant carriers in 500k 7
Annotation of predicted loss-of-function (LOF) variants 7
Characterization and visual validation of LOF variants 8
Concordance between WES, array and imputed genotypes 11
Methods for ACMG59 medically actionable variant survey 12
Definition of UK Biobank phenotypes 13
Methods for LOF Burden Association Analysis 14
Methods for single variant LOF Association Analysis 15
Methods for Replication and Follow-up 15
Somatic Variant Identification 16
Software and Code Version and Web Link Information 16
Methods References 18
Geisinger Regeneron DiscovEHR Collaboration Banner and Contribution Statement 21
Regeneron Genetics Center Banner and Contribution Statements 21

Institutional review and oversight

The Ethics Advisory Committee (EAC) is steward, monitor, and reporter for the UK Biobank Ethics and Governance Framework (EGF). The external ethics committees and relevant bodies approved and provided oversight of the scientific protocol of the UK Biobank project. This work has been conducted using the UK Biobank application 26041.

Informed consent

All participants in the UK Biobank provided informed consent.

WES sample preparation and sequencing

Genomic DNA samples normalized to approximately 16 ng/ul were transferred to the Regeneron Genetics Center from the UK Biobank in 0.5ml 2D matrix tubes (Thermo Fisher Scientific) and stored in an automated sample biobank (LiCONiC Instruments) at -80°C prior to sample preparation. One sample had insufficient DNA for sequencing. Exome capture was completed using a high-throughput, fully-automated approach developed at the Regeneron Genetics Center. Briefly, DNA libraries were created by enzymatically shearing 100ng of genomic DNA to a mean fragment size of 200 base pairs using a custom NEBNext Ultra II FS DNA library prep kit (New England Biolabs) and a common Y-shaped adapter (Integrated DNA Technologies) was ligated to all DNA libraries. Unique, asymmetric 10 base pair barcodes were added to the DNA fragment during library amplification with KAPA HiFi polymerase (KAPA Biosystems) to facilitate multiplexed exome capture and sequencing. Equal amounts of sample were pooled prior to overnight exome capture, approximately 16 hours, with a slightly modified version of IDT's xGen probe library; supplemental probes were added to capture regions of the genome well-covered by a previous capture reagent (NimbleGen VCRome) but poorly covered by the standard xGen probes (design bed file available by request). In total, n=38,997,831 bases were included in the targeted regions. Captured fragments were bound to streptavidin-coupled Dynabeads (Thermo Fisher Scientific) and non-specific DNA fragments removed through a series of stringent washes using the xGen Hybridization and Wash kit according to the manufacturer's recommended protocol (Integrated DNA Technologies). The captured DNA was PCR amplified with KAPA HiFi and quantified by qPCR with a KAPA Library Quantification Kit (KAPA Biosystems). The multiplexed samples were pooled and then sequenced using 75 base pair paired-end reads with two 10 base pair index reads on the Illumina NovaSeq 6000 platform using S2 flow cells.

Sequence alignment, variant identification, and genotype assignment

Upon completion of sequencing, raw data from each Illumina NovaSeq run was gathered in local buffer storage and uploaded to the DNAnexus platform[59] for automated analysis. After upload was complete, analysis began with the conversion of CBCL files to FASTQ-formatted reads and assigned, via specific barcodes, to samples using the bcl2fastq conversion software (Illumina Inc., San Diego, CA). Sample-specific FASTQ files, representing all the reads generated for that sample, were then aligned to the GRCh38 genome reference with BWA-mem[60]. The resultant binary alignment file (BAM) for each sample contained the mapped reads' genomic coordinates, quality information, and the degree to which a particular read differed from the reference at its mapped location. Aligned reads in the BAM file were then evaluated to identify and flag duplicate reads with the Picard[61] MarkDuplicates tool, producing an alignment file (duplicatesMarked.BAM) with all potential duplicate reads marked for exclusion in downstream analyses.

GVCF files, including variant calls, were then produced on each individual sample using the WeCall variant caller[62], identifying both SNVs and INDELs as compared to the reference. Additionally, each GVCF file carried the zygosity of each variant, read counts of both reference & alternate alleles, genotype quality representing the confidence of the genotype call, and the overall quality of the variant call at that position.

Upon completion of variant calling, individual sample BAM files were converted to fully lossless CRAM files using samtools[63]. Metric statistics were captured for each sample to evaluate capture, alignment, insert size, and variant calling quality, using Picard[61], bcftools[64], and FastQC[65].

Following completion of sample sequencing, samples showing disagreement between genetically-determined and reported sex (n=15), high rates of heterozygosity/contamination (D-stat > 0.4) (n=7), low sequence coverage (less than 85% of targeted bases achieving 20X coverage) (n=1), or genetically-identified sample duplicates (n=14), and WES variants discordant with genotyping chip (n=9) were excluded. Six samples failed quality control in multiple categories, resulting in 38 individuals being excluded. The remaining 49,960

samples were then used to compile a project-level VCF (PVCF) for downstream analysis. The PVCF was created using the GLnexus joint genotyping tool[66]. Care was taken to carry all homozygous reference, heterozygous, homozygous alternate, and no-call genotypes into the project-level VCF. An additional filtered PVCF, '*Goldilocks'* (GL), was also generated. In the filtered GL PVCF, any SNV genotype with read depth less than seven reads (DP < 7) was changed to a no-call. After the application of the DP genotype filter, only SNV variant sites that met at least one of the following two criteria were retained: 1) at least one heterozygous variant genotype with allele balance ratio greater than or equal to 15% (AB >= 0.15); 2) at least one homozygous variant genotype. The same filtering was applied to INDEL variants but with an INDEL depth filter of DP < 10 and an INDEL allele balance cutoff of AB >= 0.20. Multi-allelic variant sites in the PVCF file were normalized by left-alignment and represented as bi-allelic.

Phenotype definition

ICD10-based cases required one or more of the following: a primary diagnosis or ≥2 secondary diagnosis in in-patient Health Episode Statistics (HES) records. ICD10-based excludes had ≥1 primary or ≥2 secondary diagnosis in the code range. ICD10-based controls were defined as those individuals that were not cases or excluded. Inpatient ICD103D codes per patient provided in Table 1 reflect rolling up ICD104D inpatient codes to ICD103D codes. Custom phenotype definitions included one or more of the following: ICD-10 diagnosis, self-reported illness from verbal interview and physician-diagnosed illness from online-follow-up, touchscreen information. Quantitative measures (e.g. physical measures, blood counts, cognitive function tests, imaging derived phenotypes) were downloaded from UKB repository and spanned one or more visits. In total, data for 3,390 field IDs were downloaded from UKB repository. We selected ~1,225 field IDs for WES association tests based on sample size and preliminary genetic utility. These field IDs expanded to 1,073 binary traits with case count ≥50 and 669 quantitative traits for testing as dependent variables in WES association analyses (Supplementary Table 24).

Extrapolation of genes with LOF variant carriers in 500k

To estimate the number of genes with heterozygous LOF carriers in WES in 500k individuals, we fit a mixture model of beta binomial distributions to the observed number of heterozygous LOF carriers per gene. The mixture model is of the form $P(X,N) = \sum_i \pi_i * BetaBinomial(X, N, \alpha_i, \beta_i)$, where n is the number of LoF carriers, N is the number of sequenced samples, π describes mixing proportions for the beta-binomial components, α and β are the beta-binomial shape parameters, such that $\alpha/(\alpha+\beta)$ defines the mean of the distribution (probability of LOF per gene per individual), and $\alpha+\beta$ defines the spread of the distribution (how similar are probabilities between genes). Model parameters were estimated from LOF counts per autosomal gene in 46,911 European ancestry exomes by a likelihood expectation maximization algorithm. The best fit of the model across a range of beta-binomials was five, as measured by the Akaike information criterion. The best fit model was then used to predict the number of genes with various numbers of heterozygous carriers in 50k, and 500k individuals. (Fig. 2, Supplementary Table 25).

Annotation of predicted loss-of-function (LOF) variants

We annotated variants using SnpEff[67] and gene models from Ensembl[68] Release 85. We obtained a comprehensive and high-quality transcript set for protein coding regions which included all protein coding transcripts with an annotated Start and Stop codon from the Ensembl gene models. Variants annotated as stop_gained, start_lost, splice_donor, splice_acceptor, stop_lost and frameshift are considered predicted LOF variants.

A recent large-scale study of genetic variation in 141,456 individuals, gnomAD, provides a catalog of LOF variants[69]. A direct comparison to this data is difficult due to numerous factors such as differences in exome sequencing capture platforms, variant calling algorithms, annotation and number of individuals. Additionally,

the geographic distribution of ascertainment (and thus genetic diversity) in the Non-Finnish Europeans (NFE) subset of gnomAD may be larger than that of UK Biobank participants of European ancestry with WES in this report. Nevertheless, we annotated the gnomAD exome sites labeled as “PASS” from gnomAD r2.1 using our annotation pipeline. Data from gnomAD were lifted over to HG38 using Picard LiftoverVcf. We obtain 514,325 LOFs in the autosomes of 125,748 exomes. In comparison, 515,326 LOFs are reported in gnomAD including exome sequence and 15,708 genomes. Further, we subset the gnomAD data to NFE restricted to variants with $MAF_{NFE} < 1\%$ (Supplementary Table 26).

Identifying LOF variants based on the sequence is straight-forward. However, inferring their functional impact is not. Therefore, sequence-based LOF annotations can have annotations artifacts which can artificially inflate the number of LOFs. To control for annotation artifacts, we annotated LOFs using stringent criteria. RGC stringent LOF variants include stop gained, frameshift, splice donor and acceptor variants that affect the canonical isoform of the gene. The canonical transcripts were defined using the Ensembl definition (http://useast.ensembl.org/Help/Glossary?id=346). We also removed splice donor and acceptor variants which fall outside of the coding regions or in introns which don’t start with GT and end with AG similar to the approach used in the gnomAD study. Furthermore, we remove stop gained and frameshift variants which fall in the last 5% of the resulting protein and are predicted to escape nonsense mediated decay according to the 50 base pair rule[70].

Characterization and visual validation of LOF variants

Among LOFs observed in either dataset, 93.3% (n=188,345) were unique to WES and absent in the imputed sequence data. We observed 9,771 LOFs present in both datasets, meaning that only 4.9% of the 198,116 LOFs identified by WES were present in the imputed sequence. Since LOFs are especially informative for human genetics and medical sequencing studies, this enhancement clearly emphasizes the value of exome sequencing.

We also noted that amongst all the 515,991 imputed coding variants, 25.9% of them were not observed in the exome sequence data; a large portion of these will similarly suffer from poor imputation accuracy as observed in WES and imputed sequence concordance (Supplementary Fig. 4). As expected, common variants across functional prediction classes were more likely to be captured by both WES and imputed sequence, whereas rare variants were more likely unique to WES (Supplementary Table 5). As an expected result of purifying selection, we observed that lower frequency variants were predicted to be more deleterious as measured by CADD[71] score distributions in both datasets (Supplementary Fig. 3b). Interestingly, among rare variants, those identified by WES were typically classified as more deleterious – likely because rare variants that can be imputed may often be common in other populations even when rare in UKB.

We know that singleton variants which are the only predicted LOF variant seen in a given gene will be enriched for false positive calls. Thus, to improve the accuracy in our estimates of the number of LOF carrying genes, we performed visual validation on all 604 singletons that were the only LOF variant in their respective gene.

We interrogated these singleton LOF variants within IGV[72] by reviewing the CRAM read stacks. Various criteria can be used to make determinations of variant status (here we simply classified variants as either likely real or likely false) and are subjective depending on the reviewer. Characteristics available for review (described below) include, but are not limited to, mapping quality (MQ), depth (DP), allele balance (AB), read tiling, read strandedness, variant position within reads, duplicate read status, insert size, and mate pair consensus.

- MQ - While an MQ of 60 is best, there are many real variants called in low mappability regions. So, variants with a preponderance of MQ 0 reads are considered to be likely false, as are variants without at least one MQ 60 read supporting the call. Variants are generally considered suspect where there is little read support with unique alignments, tools like BLAT[73] can be used to confirm unique alignments for questionable reads.

- DP – Low-end depth cutoffs for our filtered variants are 7 reads for SNPs and 10 reads for INDELs. However, reviewers are cautious of sites with significant read depth, where depth may be due to homologous or repetitive sequence and will likely have a lower MQ reflecting secondary mapping sites. This can be validated by viewing the region in UCSC's Genome Browser's[74] available RepeatMasker track, but generally sites at the extremes of coverage are considered suspect.
- AB - In our Goldilocks filtered data, AB is at a site-level and is only one-sided. AB cutoffs for SNPs is 15/85 and 20/80 for INDELs. Variants outside this cutoff are considered suspect, and poor allele balance is strongly associated with likely false variants.
- Read Tiling/Coverage – This is different than depth. There ought to be even and consistent tiling of reads across the targets within the capture design. Variants that fall in regions with poor tiling often come at the edges of target regions and are likely to have issues with read strand bias.
- Read strandedness – We treat sites with a bias to one read direction as suspect. Again, using caution at edges of targets where the data is more prone to reads from only one strand, but strandedness at the edge of a capture target should be consistent between alt and ref allele reads, and properly overlap the target region.
- Variant Position – The ends of reads are error prone, so variants only seen in the ends of reads are suspect.
- Duplicate Read Status – Variants whose evidence comes primarily from reads sharing the same or nearly the same start and end points (particularly in low MAPQ regions) are considered suspect.
- Insert Size – The insert size distribution in this data is quite consistent, and generally should be consistent with templates on the order of 140-240bp long. Variants supported by read-pairs with wildly different or inconsistent insert size are considered suspect.
- Mate Pair Consensus – Where a read's mate pair overlaps, both reads should have the same sequence, and variants with evidence of inconsistent mate-pair consensus are suspect.

While visual validation is an inherently subjective process, visual validation using these criteria has been shown to correlate will with Sanger validation. For the review of this data two analysts worked independently and cross-validated a variety of the most questionable sites. Of the singleton LOF variants that were reviewed (n=604), with likely false variants (n=128) denoted with VV=0, comprising 21.2% of the variants that were reviewed. 81.25% of indels (208 of the 256 indels) and 77.01% of SNPs (268 of the 348 SNPs) passed visual read stack review (Supplementary Table 27).

When performing visual validation on homozygous reference exome calls (as with the comparison between discordant imputed calls and exome data), the reads were examined for any evidence of variation by looking for any reads supporting the called allele, for low quality alternate allele bases, and for mis-aligned, multi-nucleotide polymorphism, or soft-clipped reads containing alternate alleles at the location of the variant call. Evidence of the called allele were accounted for separately from evidence of any alternate allele as the former represents a possible undercall in the exome, while the latter is likely a misrepresentation of the variation in the region by either the imputation, exome, or both.

Concordance between WES, array and imputed genotypes

We first restricted to 46,806 European ancestry individuals that were overlapping between all three (exome, array, and imputed) datasets and had <10% missing genotype rate in both exome and array data. All datasets were restricted to autosomal variants. We excluded exome and array variants that had a HWE P-value $< 10^{-15}$, variant-level missingness >10%, and MAC<1. We excluded imputed variants with info score < 0.3 and MAF = 0. We used PLINK2 to export the filtered genotypes or dosages into transposed raw format text files. For each pair of files (exome/array and exome/imputed) we iterated through the overlapping variants and generated the Pearson R^2 correlation coefficient for all non-missing genotypes. Mean R^2 values were estimated in bins by their minor allele frequency in the filtered exome data.[75,76].

Methods for ACMG59 medically actionable variant survey

We compiled two lists of known pathogenic (P) variants reported in the ClinVar and HGMD (Human Gene Mutation Database) databases in all the ACMG59 recommended genes (Supplementary Fig. 8):

1. A high-confidence (Strict set) conservative list of 315 variants classified as "Pathogenic" or "Likely Pathogenic" with no conflicting interpretations based on stringent review and assertion criteria for clinical significance (≥2stars) was assembled from the NCBI ClinVar database. The full ClinVar dataset of variants and their corresponding classifications was downloaded from: ftp://ftp.ncbi.nlm.nih.gov/pub/clinvar/vcf_GRCh38/archive_2.0/2018/clinvar_20180429.vcf.gz
2. A comprehensive list of 1,213 pathogenic variants (Broad set) compiled from variants reported in the HGMD and ClinVar databases. This set is a union of all high-confidence disease-causing "DM" variants from HGMD (2017-12-19 version) and ClinVar "Pathogenic" and "Likely Pathogenic" without conflicting interpretations variants. From this set, variants that had discordant pathogenic annotations between ClinVar and HGMD were removed.

In addition to pathogenic variants, for both the Strict and Broad sets, we also included LOF variants in the 45 genes where, according to the ACMG recommendations[77,78], protein truncation is known to be the disease-causing mechanism and are therefore classified as likely pathogenic (LP) variants. Thus, this category also includes predicted LOF variants that may be present in HGMD but not reported in ClinVar and Pathogenic/Likely Pathogenic LOF variants in ClinVar that did not meet the assertion criteria (≥2 stars) for the Strict set of variants.

A total of 564 candidate medically actionable variants were identified within the Strict P + LP dataset. Of these, 20 variants were identified as failing the allele balance threshold of at least 20% of reads calling the alternate allele in at least one sample. All variants in the Strict dataset were visually inspected by expert curators using the Integrative Genomics Viewer (IGV). Five of the 20 variants that failed the allele balance threshold were confirmed to be of high quality albeit with allele balance ratios between 15-19%. Additionally, 7 of the 20

variants were deemed “unclear” with good quality but few (2-3) reads only calling the alternate allele; whereas 8 of the 20 variants failed visual confirmation likely representing sequence and/or alignment errors with several occurring in the vicinity of poly-A tracts and low complexity regions. Of note, rs587779193 [hg38.chr2:47414419(G>T)] a 3 star “Likely Pathogenic” splicing variant in *MSH2* reported in ClinVar was excluded from the dataset as it occurred in the vicinity of an indel failing the allele balance threshold adjacent to a low-complexity region that is prone to misalignment and consequently a likely error. Of the 16 variants that failed visual validation, 9 were LP indels. We identified 548 “Strict” known pathogenic (P, n=315) and likely pathogenic (LP, n=233) variants in the 49,960 UKB participants with WES (Supplementary Table 28). This set affects 992 non-redundant individuals (2.0% of cohort). About 60% of the variants identified were single nucleotide polymorphisms (N=328), whereas 40% were indels (N=220). Additionally, we identified and visually validated 10 complex alleles, 6 of which were dinucleotide substitutions and 4 were complex indel variants in *cis*.

For the “Broad” category, we made a union dataset of pathogenic and high confidence disease-causing variants from ClinVar and HGMD respectively along with the LP LOF set in the ACMG59 genes. Variants labelled “Benign”/“Likely Benign” or with conflicting interpretations of pathogenicity in ClinVar, or annotated as low confidence in HGMD, were removed. This resulted in 1,402 candidate medically actionable variants in 3,866 carriers (7.74% of cohort). Overall, the higher estimate of potentially actionable findings using a “Broad” set of pathogenic variants partly reflects the increased number of “disease-causing” variants reported in HGMD that have been proven to be not pathogenic upon reassessment and identification in a large number of unaffected carriers[79-82].

Definition of UK Biobank phenotypes

Quantitative measures and clinical outcomes were extracted from phenotype data available through the UK Biobank Data Showcase using definitions included in Supplementary Table 29.

Methods for LOF Burden Association Analysis

We performed burden tests of association for rare predicted LOFs within 49,960 individuals of European ancestry with WES. For each gene region as defined by Ensembl[68]. LOFs with MAF ≤ 0.01 were collapsed such that any individual that is heterozygous for at least one LOF in that gene region is considered heterozygous, and only individuals that carry two copies of the same LOF are considered homozygous. We did not phase rare variants, and so compound heterozygotes are not considered in this analysis. We additionally performed LOF burden analysis in the full 500k imputed dataset as well as a 50k imputed dataset down-sampled to the same set of individuals with WES. For these data, imputed LOFs were first converted to hardcalls using PLINK v2.0 (no imputation quality threshold, alternate allele dosage must be within 0.1 of nearest hardcall to be non-missing) and then collapsed exactly as described for WES.

For each gene region, 672 rank-based inverse normal transformed (RINT) quantitative measures (including all subjects and sex-stratified models) with ≥5 individuals with non-missing phenotype information were assessed using an additive mixed model implemented in BOLT-LMM v2[83]. Prior to normalization, traits were first transformed as appropriate (log10, square) and adjusted for a standard set of covariates including age, sex, study site, first four principal components of ancestry, and in some cases BMI and/or smoking status. Data-points greater than five median absolute deviations from the median were excluded as outliers prior to normalization. 1,059 discrete outcomes (including all subjects and sex-stratified models) with ≥ 50 cases were assessed with covariate adjustment for age, sex and first four principle components of ancestry using a generalized mixed model implemented in SAIGE[84]. For each quantitative and discrete trait included in the analysis, only gene regions in which > 3 LOF carriers with non-missing phenotype and covariate information were evaluated.

We systematically defined positive controls using a two-step approach. First, we annotated each gene for relevant disease, trait, biological, or functional evidence using publicly available resources including OMIM[85], NCBI MedGen, and the NHGRI-EBI GWAS catalogue[86]. For those genes with supporting evidence from at

least one source, we then manually curated NCBI PubMed to verify the relationship between the trait and LOF variants in the gene of interest. Genes with locus-level support for the trait of interest or related phenotype(s) in the GWAS catalog but lacking clear supporting evidence for a LOF association are reported herein as novel LOF associations.

Methods for single variant LOF Association Analysis

We performed single variant association analysis using the same methods as described in the methods section for burden association analysis. For gene-trait associations with $p<10^{-7}$ (Table 3 and Supplementary Table 12), we calculated single variant association statistics with the phenotype of interest for all LOFs included in the burden test that are observed with a minor allele count ≥5 in the European ancestry individuals with WES (Supplementary Table 30).

Methods for Replication and Follow-up

For all novel LOF associations (Table 3), we aimed to replicate the observed association in European ancestry individuals of the Geisinger-Regeneron DiscovEHR study[87]. Gene-sets for LOF burden analysis were created as previously described. For replication of quantitative traits associations, analysis was completed using linear regression with covariate adjustment for age, age-squared, sex, and first four principal components of ancestry implemented in PLINK v1.9[88]. For replication of discrete outcomes associations, analysis was completed using Firth-penalized logistic regression with covariate adjustment for age, age-squared, sex, and first four principal components of ancestry implemented in MMAP (https://mmap.github.io/). All analyses in DiscovEHR were completed in two separate batches (60k and 30k data freezes) and subsequently meta-analyzed using PLINK v1.9.

For follow-up of *MEPE* rs753138805, we leveraged data from the full UK Biobank 500k imputed dataset and the Nord-Trøndelag Health Study (HUNT)[89] using phenotypes described in Supplementary Table 31. Analyses

in the 500k imputed dataset were performed as described in "Methods for LOF Burden Association Analysis." All analyses in HUNT was completed using SAIGE.

Somatic Variant Identification

To evaluate the extent to which Clonal Hematopoiesis of Indeterminate Potential (CHIP) impacted reported association results, somatic variant calling the UKB 50k exome dataset was carried out using GATK Mutect2 (version 4.1.4.0) using GATK4 best practices as of February 2020. A panel of normals (PoN) was first created using 6,000 whole exomes from the Regeneron Geisinger DiscovEHR collaboration from individuals < 30 years of age in tumor-only mode. Mutect2 was then run to call each individual UKB 50k exome sample against this PoN as well as a population VCF of common and rare germline variants (125,748 exomes gnomAD V2). All individual mutect2 somatic calls were merged into a single population vcf file using bcftools (Version 1.9-255-g3516abc) and subset to known CHIP driver genes.

Software and Code Version and Web Link Information

Algorithms and software used for data collection and analysis are refered to in the text, including citations. All software, version information, and web links are included below and in the Software and code section of the Reporting Summary.

Data transfer from sequencing machine to DNAnexus

-Upload Agent v1.5.30 https://wiki.dnanexus.com/Downloads#Upload-Agent

Single-sample processing, all in DNAnexus

-Conversion of sequencing data in BCL format to FASTQ format and the assignments of paired-end sequence reads to samples based on 10-base barcodes; bcl2fastq v2.19.0

https://support.illumina.com/sequencing/sequencing_software/bcl2fastq-conversion-software.html -Read alignment; bwa 0.7.17 http://bio-bwa.sourceforge.net

-Duplicate marking, stats gathering; picard v1.141 https://broadinstitute.github.io/picard/

-SAM/BAM/CRAM file generation and manipulation; samtools v1.7 http://www.htslib.org

-Variant calling; WeCall v1.1.2 https://github.com/Genomicsplc/wecall

-VCF file manipulation and index generation; bcftools v1.7 http://www.htslib.org, bgzip/tabix v1.7 http://www.htslib.org

-Multi-threaded file compression and decompression; pigz v2.3.4 https://zlib.net/pigz/

<u>Generation of "freeze" data</u>

-Joint genotyping to generate project-level VCF (pVCF) files; GLnexus v0.4.0 https://github.com/dnanexus-rnd/GLnexus -Generation of variant representations in PLINK format; PLINK v1.90b3.37 https://www.cog-genomics.org/plink2/

-Ancestry predictions, IBD (Identity-by-descent) estimate, and pedigree reconstruction; PLINK v1.90b3.37 https://www.cog- genomics.org/plink2/, PRIMUS https://primus.gs.washington.edu/primusweb/

<u>Data analysis</u>

-Single variant and burden tests for quantitative traits; BOLT-LMM_v2.3.2 https://data.broadinstitute.org/alkesgroup/BOLT-LMM/ -Single variant and burden tests for binary outcomes; SAIGE_v0.29.1 https://github.com/weizhouUMICH/SAIGE

-GHS quantitative results; PLINK v1.90b3.38 64-bit (7 Jun 2016) https://www.cog-genomics.org/plink/1.9/

-Various, including GHS meta analyses; PLINK v1.90b3.45 64-bit (13 Jan 2017) https://www.cog-genomics.org/plink/1.9/

-Imputed sequence conversion; PLINK v2.00a2LM AVX2 Intel (31 Mar 2018) https://www.cog-genomics.org/plink/2.0/

Methods References

59 Reid, J. G. *et al.* Launching genomics into the cloud: deployment of Mercury, a next generation sequence analysis pipeline. *BMC Bioinformatics* **15**, 30, doi:10.1186/1471-2105-15-30 (2014).

60 Li, H. & Durbin, R. Fast and accurate short read alignment with Burrows-Wheeler transform. *Bioinformatics* **25**, 1754-1760, doi:10.1093/bioinformatics/btp324 (2009).

61 BroadInstitute. *Picard*, <http://picard.sourceforge.net> (2018).

62 GenomicsPLC. *weCall*, <https://github.com/Genomicsplc/wecall> (2018).

63 Li, H. *et al.* The Sequence Alignment/Map format and SAMtools. *Bioinformatics* **25**, 2078-2079, doi:10.1093/bioinformatics/btp352 (2009).

64 Danecek, P., McCarthy, S. & Marshall., J. *SAMtools*, <http://samtools.github.io/bcftools> (2018).

65 Andrews, S. *FastQC*, <http://www.bioinformatics.babraham.ac.uk/projects/fastqc> (2014).

66 Lin, M. F. *et al.* GLnexus: joint variant calling for large cohort sequencing. *bioRxiv*, doi:10.1101/343970 (2018).

67 Cingolani, P. *et al.* A program for annotating and predicting the effects of single nucleotide polymorphisms, SnpEff: SNPs in the genome of Drosophila melanogaster strain w1118; iso-2; iso-3. *Fly (Austin)* **6**, 80-92, doi:10.4161/fly.19695 (2012).

68 Zerbino, D. R. *et al.* Ensembl 2018. *Nucleic Acids Res* **46**, D754-D761, doi:10.1093/nar/gkx1098 (2018).

69 Karczewski, K. J. *et al.* Variation across 141,456 human exomes and genomes reveals the spectrum of loss-of-function intolerance across human protein-coding genes. *bioRxiv*, doi:10.1101/531210 (2019).

70 Isken, O. & Maquat, L. E. Quality control of eukaryotic mRNA: safeguarding cells from abnormal mRNA function. *Genes Dev* **21**, 1833-1856, doi:10.1101/gad.1566807 (2007).

71 Rentzsch, P., Witten, D., Cooper, G. M., Shendure, J. & Kircher, M. CADD: predicting the deleteriousness of variants throughout the human genome. *Nucleic Acids Res* **47**, D886-D894, doi:10.1093/nar/gky1016 (2019).

72 Thorvaldsdottir, H., Robinson, J. T. & Mesirov, J. P. Integrative Genomics Viewer (IGV): high-performance genomics data visualization and exploration. *Brief Bioinform* **14**, 178-192, doi:10.1093/bib/bbs017 (2013).

73 Kent, W. J. BLAT--the BLAST-like alignment tool. *Genome Res* **12**, 656-664, doi:10.1101/gr.229202 (2002).

74 Kent, W. J. *et al.* The human genome browser at UCSC. *Genome Res* **12**, 996-1006, doi:10.1101/gr.229102 (2002).

75 Bycroft, C. *et al.* The UK Biobank resource with deep phenotyping and genomic data. *Nature* **562**, 203-209, doi:10.1038/s41586-018-0579-z (2018).

76 McCarthy, S. *et al.* A reference panel of 64,976 haplotypes for genotype imputation. *Nat Genet* **48**, 1279-1283, doi:10.1038/ng.3643 (2016).

77 Green, R. C. *et al.* ACMG recommendations for reporting of incidental findings in clinical exome and genome sequencing. *Genet Med* **15**, 565-574, doi:10.1038/gim.2013.73 (2013).

78 Kalia, S. S. *et al.* Recommendations for reporting of secondary findings in clinical exome and genome sequencing, 2016 update (ACMG SF v2.0): a policy statement of the American College of Medical Genetics and Genomics. *Genet Med* **19**, 249-255, doi:10.1038/gim.2016.190 (2017).

79 Wright, C. F. *et al.* Assessing the Pathogenicity, Penetrance, and Expressivity of Putative Disease-Causing Variants in a Population Setting. *Am J Hum Genet*, doi:10.1016/j.ajhg.2018.12.015 (2019).

80 Wang, J. & Shen, Y. When a "disease-causing mutation" is not a pathogenic variant. *Clin Chem* **60**, 711-713, doi:10.1373/clinchem.2013.215947 (2014).

81 Bell, C. J. *et al.* Carrier testing for severe childhood recessive diseases by next-generation sequencing. *Sci Transl Med* **3**, 65ra64, doi:10.1126/scitranslmed.3001756 (2011).

82 Improving databases for human variation. *Nat Methods* **13**, 103, doi:10.1038/nmeth.3762 (2016).

83 Loh, P. R., Kichaev, G., Gazal, S., Schoech, A. P. & Price, A. L. Mixed-model association for biobank-scale datasets. *Nat Genet* **50**, 906-908, doi:10.1038/s41588-018-0144-6 (2018).

84 Zhou, W. *et al.* Efficiently controlling for case-control imbalance and sample relatedness in large-scale genetic association studies. *Nat Genet* **50**, 1335-1341, doi:10.1038/s41588-018-0184-y (2018).

85 Amberger, J. S., Bocchini, C. A., Schiettecatte, F., Scott, A. F. & Hamosh, A. OMIM.org: Online Mendelian Inheritance in Man (OMIM(R)), an online catalog of human genes and genetic disorders. *Nucleic Acids Res* **43**, D789-798, doi:10.1093/nar/gku1205 (2015).

86 MacArthur, J. *et al.* The new NHGRI-EBI Catalog of published genome-wide association studies (GWAS Catalog). *Nucleic Acids Res* **45**, D896-D901, doi:10.1093/nar/gkw1133 (2017).

87 Dewey, F. E. *et al.* Distribution and clinical impact of functional variants in 50,726 whole-exome sequences from the DiscovEHR study. *Science* **354**, doi:10.1126/science.aaf6814 (2016).

88 Chang, C. C. *et al.* Second-generation PLINK: rising to the challenge of larger and richer datasets. *Gigascience* **4**, 7, doi:10.1186/s13742-015-0047-8 (2015).

89 Krokstad, S. *et al.* Cohort Profile: the HUNT Study, Norway. *Int J Epidemiol* **42**, 968-977, doi:10.1093/ije/dys095 (2013).

Geisinger Regeneron DiscovEHR Collaboration Banner and Contribution Statement

All authors/contributors are listed in alphabetical order.

Lance J. Adams[1], Jackie Blank[1], Dale Bodian[1], Derek Boris[1], Adam Buchanan[1], David J. Carey[1], Ryan D. Colonie[1], F. Daniel Davis[1], Dustin N. Hartzel[1], Melissa Kelly[1], H. Lester Kirchner[1], Joseph B. Leader[1], David H. Ledbetter[1], Ph.D., J. Neil Manus[1], Christa L. Martin[1], Michelle Meyer[1], Tooraj Mirshahi[1], Matthew Oetjens[1], Thomas Nate Person[1], Christopher Still[1], Natasha Strande[1], Amy Sturm[1], Jen Wagner[1], Marc Williams[1]

Contribution: Development and validation of clinical phenotypes used to identify study participants and (when applicable) controls.

Affiliations:

1. Geisinger, Danville, PA

Regeneron Genetics Center Banner and Contribution Statements

All contributors are listed in alphabetical order.

<u>RGC Management and Leadership Team:</u>

Goncalo R. Abecasis, D.Phil.[1], Aris Baras, M.D.[1], Michael Cantor, M.D.[1], Giovanni Coppola, M.D.[1], Aris Economides, Ph.D.[1], John D. Overton, Ph.D.[1], Jeffrey G. Reid, Ph.D.[1], Alan R. Shuldiner, M.D.[1]

Contribution: All authors contributed to securing funding, study design and oversight, and review and interpretation of data and results.

Sequencing and Lab Operations:

Christina Beechert[1], Erin Brian[1], Alex DeVito[1], Caitlin Forsythe[1], Erin D. Fuller[1], Zhenhua Gu[1], Joe LaRosa[1], Michael Lattari[1], Alexander Lopez[1], Kia Manoochehri[1], Justin Marcovici[1], Manasi Pradhan[1], John D. Overton, Ph.D.[1], Thomas D. Schleicher[1], Maria Sotiropoulos Padilla[1], Karina Toledo[1], Emelia Weihenig[1], Louis Widom[1], Sarah E. Wolf[1], Ricardo H. Ulloa[1]

Contribution: Performed and are responsible for sample genotyping and exome sequencing, conceived and are responsible for laboratory automation, and responsible for sample tracking and the library information management system.

Genome Informatics:

Xiaodong Bai, Ph.D.[1], Suganthi Balasubramanian, Ph.D.[1], Leland Barnard, Ph.D.[1], Andrew Blumenfeld[1], Boris Boutkov[1], Yating Chai, Ph.D.[1], Gisu Eom[1], Lukas Habegger, Ph.D.[1], Young Hahn[1], Alicia Hawes[1], Shareef Khalid[1], Olga Krasheninina[1], Rouel Lanche[1], Adam Mansfield[1], Evan K. Maxwell, Ph.D.[1], Mona Nafde[1], Sean O'Keeffe, Ph.D.[1], John Penn[1], Ayesha Rasool[1], William Salerno, Ph.D.[1], Jeffrey C. Staples, Ph.D.[1], Jeffrey G. Reid, Ph.D[1]

Contribution: Performed and are responsible for analysis needed to produce exome and genotype data, provided compute infrastructure development and operational support, provided variant and gene annotations and their functional interpretation of variants, and conceived and are responsible for creating, developing, and deploying analysis platforms and computational methods for analyzing genomic data.

Clinical Informatics:

Nilanjana Banerjee, Ph.D.[1], Michael Cantor, M.D.[1], Dadong Li Ph.D.[1], Fabricio Sampaio Peres Kury M.D.[1], Deepika Sharma B.H.M.S.[1], Ashish Yadav[1]

Contribution: All authors contributed to the development and validation of clinical phenotypes used to identify study participants and (when applicable) controls.

Analytical Genomics and Data Science:

Goncalo R. Abecasis, D.Phil.[1], Joshua Backman, Ph.D.[1], Mathew Barber, Ph.D.[1], Christian Benner, Ph.D.[1], Shan Chen, Ph.D.[1], Amy Damask, Ph.D.[1], Manuel Allen Revez Ferreira, Ph.D.[1], Lauren Gurski[1], Jack Kosmicki, Ph.D.[1], Alexander Li, Ph.D.[1], Nan Lin, Ph.D.[1], Daren Liu[1], Jonathan Marchini Ph.D.[1], Anthony Marcketta[1], Joelle Mbatchou, Ph.D.[1], Shane McCarthy, Ph.D.[1], Colm O'Dushlaine, Ph.D.[1], Charles Paulding, Ph.D.[1], Claudia Schurmann, Ph.D.[1], Dylan Sun[1], Cristopher Van Hout, Ph.D.[1], Kyoko Watanabe, Ph.D.[1], Bin Ye[1], Andrey Ziyatdinov, Ph.D.[1]

Contribution: Development of statistical analysis plans. QC of genotype and phenotype files and generation of analysis ready datasets. Development of statistical genetics pipelines and tools and use thereof in generation of the association results. QC, review and interpretation of result. Generation and formatting of results for manuscript figures.

Therapeutic Area Genetics:

Ariane Ayer[1], Giovanni Coppola M.D.[1], Silvio Alessandro Di Gioia, Ph.D.[1], Jan Freudenberg, M.D.[1], Sahar Gelfman, Ph.D.[1], Claudia Gonzaga-Jauregui, Ph.D.[1], Nehal Gosalia, Ph.D.[1], Julie Horowitz, Ph.D.[1], Luca Lotta M.D. Ph.D.[1], Kavita Praveen, Ph.D.[1]

Contribution: Development of study design and analysis plans. Development and QC of phenotype definitions. QC, review, and interpretation of association results.

Functional Modeling:

Shek Man Chim, Ph.D. [1], Giusy Della Gatta, Ph.D.[1], Aris Economides, Ph.D.[3], Lawrence Miloscio[1], Harikiran Nistala, Ph.D.[1], Trikaldarshi Persaud[1]

Contribution: Development of *in vivo* and *in vitro* experimental biology and interpretation.

Planning, Strategy, and Operations:

Paloma M. Guzzardo, Ph.D.[4], Marcus B. Jones, Ph.D.[4], Michelle LeBlanc, Ph.D.[4], Jason Mighty, Ph.D.[4], Lyndon J. Mitnaul, Ph.D.[4]

Contribution: Contributed to the management and coordination of all research activities, planning and execution, managed the review of the project.

Affiliations:

1. Regeneron Genetics Center, Tarrytown, NY USA

4. Regeneron Pharmaceuticals, Tarrytown, NY USA

Demographic and Clinical Characteristics	UKB 50k WES Participants	UKB 500k Participants
N	49,960	502,543
Female, n(%)	27,243 (54.5)	273,460 (54.4)
Age at assessment, years (1st-3rd Quartiles) a	58 (45-71)	58 (45-71)
Body mass index, kg/m^2 (1st-3rd Quartiles) a	26 (21-31)	26 (21-31)
Number of imaged participants (%) a	12,075 (24.1)[b]	21,407 (4.3)[bc]
Number of current/past smokers, n(%) a	17,515 (35.0)	216,482 (43.1)
Townsend Deprivation Index (1st-3rd Quartiles) a	-2.0 (-6.1, -2.1)	-2.1 (-6.2, -1.9)
Inpatient ICD10 3D codes per patient (1st-3rd Quartiles)	5 (2,9)	5 (2,9)
Patients with ≥1 inpatient ICD10 diagnoses, n(%)	42,066 (84.2)	391,983 (78.0)
**Genetic Ancestry Assignment**[d]		
African (%)	1.49	1.24
East Asian (%)	0.54	0.51
European (%)	93.6	94.5
**Cardiometabolic phenotypes**		
Coronary Disease, n(%)	3,340 (6.7)	35,879 (7.1)
Heart Failure, n(%)	300 (0.6)	4,399 (0.8)
Type 2 Diabetes, n(%)	1,541 (3.0)	17,261 (3.4)
**Respiratory and immunological phenotypes**		
Asthma, n(%)	8,250 (16.5)	68,149 (13.5)
COPD, n(%)	741 (1.4)	7,438 (1.4)
Rheumatoid Arthritis, n(%)	710 (1.4)	7,337 (1.4)
Inflammatory Bowel Disease n(%)	543 (1.0)	5,783 (1.1)
**Neurodegenerative phenotypes**		
Alzheimer's Disease, n(%)	13 (0.05)	320 (0.06)
Parkinson's Disease, n(%)	65 (0.13)	1,043 (0.21)
Multiple Sclerosis, n(%)	126 (0.25)	1,352 (0.26)
Myasthenia Gravis, n(%)	14 (0.02)	217 (0.04)
**Oncology phenotypes**		
Breast Cancer in Females, n(% in Females)	1,657 (6.1)	16,772 (6.1)
Ovarian Cancer, n(% in Females)	162 (0.6)	1,777 (0.6)
Pancreatic Cancer, n(%)	602 (1.2)	4,611 (0.9)
Prostate Cancer, n(% in Males)	848 (3.7)	8,855 (3.9)
Melanoma, n(%)	598 (1.1)	5,715 (1.1)
Lung Cancer, n(%)	172 (0.3)	2,581 (0.5)
Colorectal Cancer, n(%)	368 (0.7)	3,971 (0.8)
Cutaneous squamous cell carcinoma, n(%)	1,316 (2.6)	12,969 (2.6)
**Enhanced measures** [a]		
Hearing test, n(%)	40,546 (81.1)	167,011 (33.2)
Pulse Rate, n(%)	40,548 (34.2)	170,761 (33.9)
Visual Acuity Measured, n(%)	39,461 (78.9)	117,092 (23.2)
IOP measured (left), n(%)	37,940 (75.9)	111,942 (22.2)
Autorefraction, n(%)	36,067 (72.1)	105,989 (21.0)
Retinal OCT, n(%)	32,748 (65.5)	67,708 (13.4)
ECG at rest, n(%)	10,829 (27.1)	13,572 (2.1)
Cognitive Function, n(%)	9,511 (19.0)	96,362 (19.1)
Digestive Health, n(%)	13,553 (28.1)	142,310 (28.3)

Physical Activity Measurement, n(%)	10,684 (21.3)	101,117 (20.1)11

Supplementary Table 1 | Clinical characteristics in whole exome sequenced and all UK Biobank participants. Demographics and clinical characteristics of UKB 50K sequenced participants and overall 500K participants. See Supplementary Information for definition of UKB clinical phenotype definitions. Values are expressed as median (1st and 3rd quartile) or as counts (and percentages). [a]Demographic and enhanced measures counts were based on data from initial assessment visit. [b]The number of samples with exome sequencing data and at least one non-missing image derived phenotype value from data downloaded from UK Biobank in November 2018.[c]The number of samples with at least one non-missing image derived phenotype value from data downloaded from UK Biobank in November 2018. [d]Number of samples in 3 pre-defined regions of a plot of the first two genetic principal component scores, where the regions are selected to represent African, East Asian, and European ancestry (Supplementary Figure 2**).**

Frequency	Variant Type	Metric	ALL	LOF	MISSENSE	SYN
All Frequencies	snp	count	4,540,330	99,803	2,492,667	1,224,107
		median_allele_balance	0.468	0.454	0.467	0.471
		median_depth	40.6	37.1	40.4	41.5
		median_genotype_quality	687.6	604.7	687.2	717.3
		median_variant_reads	25.9	22.9	25.9	27.1
		missingness	8.8	11.5	8.1	7.2
	indel	count	212,447	98,466	0	0
		median_allele_balance	0.465	0.461	0	0
		median_depth	39.2	37.9	0	0
		median_genotype_quality	792.0	780.4	0	0
		median_variant_reads	19.8	20.8	0	0
		missingness	34.3	10.7	0	0
MAF<1%	snp	count	4,473,371	99,452	2,466,312	1,197,927
		median_allele_balance	0.467	0.454	0.466	0.470
		median_depth	40.5	37.1	40.3	41.4
		median_genotype_quality	683.1	603.8	684.0	711.0
		median_variant_reads	25.7	22.8	25.8	26.9
		missingness	8.7	11.4	8.1	7.1
	indel	count	209,153	98,121	0	0
		median_allele_balance	0.464	0.461	0	0
		median_depth	39.1	37.9	0	0
		median_genotype_quality	791.0	779.3	0	0
		median_variant_reads	19.6	20.7	0	0
		missingness	30.3	10.3	0	0

Supplementary Table 2 | Quality control metrics for functional variants. Mean of quality control measures for 4,752,777 variants in targeted regions from 49,960 individuals with WES, stratified by functional classification.

	All		Males, median (IQR)		Females, median (IQR)	
	# Variants	# Variants AAF[1]<1%	# Variants	# Variants AAF<1%	# Variants	# Variants AAF<1%
Total	247,458	244,973	569 (41)	77 (14)	837 (47)	104 (16)
Targeted Regions[2]	114,186	113,003	296 (24)	40 (9)	415 (25)	49 (10)
**Variant Type[2]**						
SNV	109,214	108,114	279 (24)	38 (9)	395 (24)	46 (10)
Indel	4,972	4,889	17 (4)	2 (2)	20 (4)	2 (2)
Multi-Allelic	12,113	11,904	33 (6)	6 (2)	50 (6)	8 (3)
**Predicted Function**						
Synonymous	31,356	30,904	121 (13)	13 (5)	172 (14)	16 (5)
Missense	59,796	59,351	96 (12)	12 (5)	139 (13)	17 (6)
LOF (any transcript)	3,670	3,659	3 (2)	1 (1)	4 (2)	1 (1)
LOF (all transcripts)	2,723	2,715	3 (1)	1 (0)	3 (1)	1 (0)
LOF (LOFTEE)	3,055	3,043	3 (1)	1 (0)	3 (1)	1 (0)

Supplementary Table 3 | Summary statistics for variants in sequenced exomes of 49,960 UKB participants on chromosome X. Counts of chromosome X variants observed across all individuals and stratified by sex (22,716 males, 27,244 females) and by type/functional class for all variants and for AAF<1% frequency. Pseudoautosomal (PAR1 and PAR2) regions were excluded in both sexes. Heterozygous genotypes in males were set to missing. All variants passed Hardy-Weinberg threshold p-value>10^{-15} (in females only), variant missingness and individual missingness <10%. [1]Chromosome X frequencies are estimated from both sexes assuming diploid females and haploid males. [2] Counts restricted to WES targeted regions.

	AAF[1]	WES	Imputed 50k	Both
LOF	All	198,116	13,561	9,771
	<0.01%	185,500	6,295	3,933
	0.01-0.1%	10,177	5,003	3,981
	0.1-1%	1,736	1,534	1,283
	1-5%	355	319	282
	>5%	198,116	13,561	9,771
Synonymous	All	1,223,633	249,263	202,210
	<0.01%	1,044,729	103,589	74,033
	0.01-0.1%	123,811	86,253	76,153
	0.1-1%	28,549	31,226	26,561
	1-5%	8,699	8,979	8,170
	>5%	1,223,633	249,263	202,210
Missense	All	2,491,290	384,406	295,699
	<0.01%	2,223,561	181,190	122,333
	0.01-0.1%	200,377	132,966	111,767
	0.1-1%	40,543	42,443	36,219
	1-5%	10,769	10,681	9,877
	>5%	2,491,290	384,406	295,699

Supplementary Table 4 | Ascertainment of variation in WES and imputed sequence. Counts of exome targeted autosomal predicted LOF, synonymous, and missense variation in 46,911 individuals of European ancestry with both WES and imputed sequence. Imputed sequence includes the same set of 46,911 individuals. [1]Counts of variants in WES and Both categories are binned by alt allele frequency (AAF) in WES. Counts in imputed 50k are binned by AAF in WES if observed in WES, otherwise by AAF in imputed sequence.

	Variants in Imputed Sequence, info > 0.3, n=49,797 Participants		Median Per Participant (IQR)	
	# Variants	# Variants MAF≤1%	# Variants	# Variants MAF≤1%
Total	62,644,999	53,296,285	3,708,914 (18,537)	39,395 (1,958)
Targeted Regions[1]	780,445	708,506	26,129 (291)	510 (46)
**Variant Type[1]**				
SNVs	767,266	697,707	25,220 (283)	498 (45)
Indels	13,179	10,799	909 (28)	13 (5)
**Functional Prediction**				
Synonymous	254,423	226,453	10,615 (133)	153 (22)
Missense	396,021	367,467	9,681 (142)	273 (29)
LOF (any transcript)	18,055	17,012	340 (17)	13 (5)
LOF (all transcripts)	7,392	7,004	90 (11)	6 (3)
LOF (LOFTEE)	12,904	12,340	169(12)	9 (5)

Supplementary Table 5 | Summary statistics for variants in imputed sequence. Counts of autosomal variants observed in n=49,797 UKB participants with WES and imputed sequence by type/functional class for all and for MAF <1% frequency. All imputed variants have imputation info score >0.3. Median count of variants and interquartile range (IQR) for all variants and for MAF <1%. [1] Counts restricted to WES targeted regions.

		# Autosomal genes containing at least N LOFs, MAF < 1%					
Zygosity	Genomic resource	1	5	10	25	50	100
Het	50k exome	17,718	15,253	12,598	7,762	4,553	2,441
	50k imputed (info > 0.3)	7,500	5,882	4,950	3,489	2,344	1,460
	500k imputed (info > 0.3)	8,724	7,711	7,267	6,539	5,847	4,916
Hom	50k exome	789	92	18	2	0	0
	50k imputed (info > 0.3)	612	50	6	0	0	0
	500k imputed (info > 0.3)	1,752	597	351	120	21	3

Supplementary Table 6 | Number of autosomal genes with heterozygous, homozygous LOF variants. Count of genes with at least the specified number of LOFs (MAF < 1%) impacting any transcript in European ancestry in approximately 50k (n=46,911 with WES and imputed sequence), and 462,427 individuals for 500k imputed sequence.

		# Autosomal genes containing at least N LOFs, MAF < 1%					
**Zygosity**	**Genomic resource**	**1**	**5**	**10**	**25**	**50**	**100**
Het	50k exome	17,718	15,253	12,598	7,762	4,553	2,441
	50k imputed (all)	7,808	5,955	5,003	3,500	2,355	1,464
	50k imputed (info > 0.3)	7,500	5,882	4,950	3,489	2,344	1,460
	50k imputed (info > 0.5)	6,348	5,226	4,490	3,260	2,229	1,408
	50k imputed (info > 0.8)	3,276	2,757	2,468	1,854	1,415	1,013
	500k imputed (all)	9,346	8,086	7,485	6,644	5,915	4,956
	500k imputed (info > 0.3)	8,724	7,711	7,267	6,539	5,847	4,916
	500k imputed (info > 0.5)	7,208	6,449	6,171	5,703	5,199	4,468
	500k imputed (info > 0.8)	3,711	3,332	3,177	2,956	2,755	2,475
Hom	50k exome	789	92	18	2	-	-
	50k imputed (all)	612	50	6	-	-	-
	50k imputed (info > 0.3)	612	50	6	-	-	-
	50k imputed (info > 0.5)	598	50	6	-	-	-
	50k imputed (info > 0.8)	478	44	6	-	-	-
	500k imputed (all)	1,755	598	351	120	21	3
	500k imputed (info > 0.3)	1,752	597	351	120	21	3
	500k imputed (info > 0.5)	1,701	586	346	120	21	3
	500k imputed (info > 0.8)	1,197	500	300	106	18	3

Supplementary Table 7 | Number of autosomal genes with heterozygous, homozygous LOF variants.

Count of genes with at least the specified number of LOFs (MAF <1%) in UK Biobank participants of European ancestry in approximately 50k (n=46,911 with WES and imputed sequence) and 500k (462,427 individuals).

Category	#Variants	% of Total Known ACMG59 Variants	#Carriers	% of individuals with reportable variants
**Pathogenic (P)**	315	4.23	692	1.39
**Likely Pathogenic (LP)**	233	-	307	0.61
**P + LP**	548	-	992	2.0[1]

Supplementary Table 8 | Medically actionable variants in ACMG59 genes in UKB participants. Of the 49,960 UKB participants with WES data, 2.0% are carriers of pathogenic (P) or likely pathogenic (LP) variants in ACMG59 v2.0 genes based on strict variant filtering criteria. LP variant counts include LOF variants passing QC criteria in ACMG59 genes that are not reported in ClinVar (≥2 star). Amongst all P+LP variants, 379 variants were observed in only one individual, 163 were observed in 2-10 individuals, and 8 were observed in >10 individuals. [1]Percent of individuals with P or LP variants is not additive, as the 2.0% represents non-redundant carriers; 9 individuals were found to have 2 medically actionable variants.

Category	#Variants	% of Total Known ACMG59Variants	#Carriers	% of individuals with reportable variants
**Broad (P)**	1213	3.8	3644	7.29
**Broad + LP**	189	-	251	0.50

Supplementary Table 9 | Variation in ACMG genes in UKB WES. UKB participants are carriers of known pathogenic (P) and likely (LP) pathogenic variants in ACMG59 v2.0 genes based on broad variant filtering criteria. Notably, the number and proportion of individuals estimated to carry a reportable variant using the broad definition (See Methods) is inconsistent with the population prevalence of Mendelian disorders.

Category	Number of Variants	% of Total Known Variants	Number of Carriers	% of individuals with Reportable Variants
**Pathogenic (P)**	540	7.31	1774	1.94
**Likely Pathogenic (LP)**	396	-	769	0.84
**Total**	936	-	2527	2.76

Supplementary Table 10 | Variation in ACMG genes in Regeneron-Geisinger DiscovEHR WES. 2.76% of GHS participants are carriers of known pathogenic (P) and likely (LP) pathogenic variants in ACMG59 v2.0 genes based on the strict variant filtering criteria compared to 2.03% in UKB (See Methods).

Group	LP #Carriers	P #Carriers	LP/P #NonCarriers
Female Breast Cancer Control	11	74	25,499
Female Breast Cancer Case	1	23	1,630
Melanoma Control	20	198	49,127
Melanoma Case	0	5	594
Ovarian Cancer Control	9	94	26,973
Ovarian Cancer Case	3	3	156
Prostate Cancer Control	7	93	21,718
Prostate Cancer Case	1	13	874
Pancreatic Cancer Control	20	194	49,128
Pancreatic Cancer Case	0	9	593
Any Cancers Control	12	136	38,159
Any Cancers Case	8	67	11,562
Any 5 Cancers Control	15	161	46,423
Any 5 Cancers Case	5	42	3,290
Female Breast Ovarian Cancers Control	8	73	25,353
Female Breast Ovarian Cancers Case	4	24	1,776
Other Cancers Control	12	137	38,275
Other Cancers Case	3	25	8,272

Supplementary Table 11 | Number of carriers and non-carriers of pathogenic variants in cancer cases and controls in UK Biobank. Number of individuals carrying *BRCA1* or *BRCA2* Likely Pathogenic (LP), known Pathogenic (P), and non-carriers of either LP or P pathogenic variants in cancer cases and controls. Analysis of 5 cancer types includes breast cancer in females, ovarian, melanoma, pancreatic and prostate cancers.

Gene	ICD10 Code, Binary Phenotype	RR|RA|AA	OR (95% CI)	WES Burden P	N SNV	Lowest P SNV	Imputed 50k Burden P
MLH1	Z85.0, Personal history of malignant neoplasm of digestive organs	Ctrl:39634|11|0 Case:319|6|0	70 (19, 250)	$8.2x10^{-11}$	12	0.87	NA[1]
PKD1	N18, Chronic kidney disease	Ctrl:46289|16|0 Case:210|6|0	86 (21, 350)	$6.4x10^{-10}$	18	NA[1]	NA[2]
TTN	I42, Cardiomyopathy	Ctrl:44255|575|0 Case:67|11|0	12 (5.2, 29)	$6.7x10^{-9}$	360	$1.2x10^{-3}$	0.016

Gene	Quantitative Phenotype	RR|RA|AA	Beta (95% CI)	WES Burden P	N SNV	Lowest P SNV	Imputed 50k Burden P
IL33	Eosinophil percentage	44859|504|0	-0.3 (-0.38, -0.21)	$2.4x10^{-11}$	11	$4.1x10^{-12}$	$2.7x10^{-11}$
IL33	Eosinophil count	44602|502|0	-0.29 (-0.37, -0.2)	$1.3x10^{-10}$	11	$2.9x10^{-11}$	$6.8x10^{-10}$
GP1BA	Mean platelet thrombocyte volume	45427|98|1	0.51 (0.32, 0.69)	$7.7x10^{-8}$	13	$3.9x10^{-5}$	NA[2]
TUBB1	Platelet distribution width	45495|31|0	1.8 (1.4, 2.2)	$1.2x10^{-22}$	18	$3.2x10^{-6}$	$5.3x10^{-3}$
TUBB1	Mean platelet thrombocyte volume	45460|28|0	0.95 (0.61, 1.3)	$3.6x10^{-8}$	18	$2.1x10^{-3}$	0.028
TUBB1	Platelet count	45388|30|0	-1.1 (-1.4, -0.72)	$2.1x10^{-9}$	18	$1.0x10^{-7}$	0.029
HBB	Red blood cell erythrocyte count	45512|4|0	3 (2, 3.9)	$1.7x10^{-9}$	10	NA[1]	0.23
HBB	Red blood cell erythrocyte distribution width	44987|4|0	2.6 (1.7, 3.6)	$7.4x10^{-8}$	10	NA[1]	0.70
KLF1	Red blood cell erythrocyte distribution width	44966|25|0	1.4 (1.1, 1.8)	$2.0x10^{-13}$	10	$5.0x10^{-10}$	0.43
KLF1	Mean corpuscular haemoglobin	45318|27|0	-1.5 (-1.9, -1.1)	$1.2x10^{-15}$	10	$4.0x10^{-12}$	0.79
KLF1	Mean corpuscular volume	45352|27|0	-1.4 (-1.8, -1)	$5.9x10^{-14}$	10	$1.4x10^{-10}$	0.66
ASXL1	Platelet distribution width	45388|100|0	0.59 (0.4, 0.78)	$1.8x10^{-9}$	46	$8.2x10^{-5}$	NA[2]
ASXL1	Red blood cell erythrocyte distribution width	44894|97|0	0.58 (0.38, 0.77)	$6.7X10^{-9}$	46	$7.5x10^{-6}$	NA[2]
CHEK2	Platelet crit	45097|299|1	0.3 (0.19, 0.41)	$9.8x10^{-8}$	38	$3.4x10^{-7}$	0.054
KALRN	Mean platelet thrombocyte volume	45296|229|1	-0.63 (-0.75, -0.51)	$5.8x10^{-24}$	20	$1.3x10^{-23}$	$1.7x10^{-20}$
COL4A4	MCR no CKD	13712|45|0	0.92 (0.62, 1.2)	$8.2x10^{-10}$	27	$9.2x10^{-8}$	0.84

Supplementary Table 12 | LOF gene burden results with previously known genetic associations. LOF gene burden association with available clinical and continuous traits in 46,876 UKB participants of European

ancestry with WES. [1]SNV/Gene did not reach threshold for association analysis (MAC ≥4). [2]No LOFs were observed in imputed sequence.

Gene	ICD10 code, Binary Phenotype	Control RR|RA|AA	Case RR|RA|AA	OR (95% CI)	P-Value
BRCA1	Z40.0, Encounter for prophylactic surgery for risk factors related to malignant neoplasms	43355|57|0	58|4|0	56 (11,290)	$1.1x10^{-6}$
BRCA2	C61, Malignant neoplasm of prostate	20272|443|1	595|30|0	2.3 (1.5,3.5)	$8.8x10^{-5}$
CALR	D47, Other neoplasms of uncertain behavior of lymphoid, hematopoietic and related tissue	46453|2|0	52|3|0	1200 (83,18000)	$2.1x10^{-7}$
COL4A4	R31, Hematuria	44271|72|0	1309|12|0	5.8 (2.8,12)	$2.1x10^{-6}$
KRT5	L90.5, Scar conditions and fibrosis of skin	45319|10|0	204|1|0	32 (2.3,440)	0.010
LDLR	I25.1, Atherosclerotic heart disease of native coronary artery	43694|27|0	1646|3|0	3.4 (1.1,10)	0.031
PALB2	C50, Malignant neoplasm of breast	45646|105|0	1100|9|0	3.7 (1.7,8.2)	$1.1x10^{-3}$
RBM20	I34.0, Nonrheumatic mitral (valve) insufficiency	44773|21|0	108|3|0	66 (11,420)	$7.2x10^{-6}$
SMAD6	M50, Cervical disc disorders	44968|47|0	118|5|0	43 (10,180)	$2.2x10^{-7}$

Gene	Quantitative Phenotype	RR|RA|AA	Beta (95% CI)	P-Value
COL6A3	Corneal resistance factor mean	35493|57|0	-0.6 (-0.86,-0.35)	$3.8x10^{-6}$
GP1BB	Platelet count	45414|4|0	-2.3 (-3.2,-1.3)	$2.7x10^{-6}$
IL17RA	Monocyte count	5298|19|0	-1.1 (-1.6,-0.69)	$5.1x10^{-7}$
JAK2	Platelet count	45393|25|0	0.86 (0.48,1.2)	$1.0x10^{-5}$
RHAG	Mean sphered cell volume	43404|19|0	-1.1 (-1.5,-0.63)	$1.8x10^{-6}$
TET2	Eosinophil count	44956|148|0	-0.43 (-0.59,-0.27)	$1.5x10^{-7}$
TMPRSS6	Mean corpuscular haemoglobin	45305|40|0	-0.78 (-1.1,-0.48)	$3.7x10^{-7}$

Supplementary Table 13 | Extended list of positive control LOF burden results in UK Biobank. LOF gene burden association with available clinical and continuous traits in 46,876 UKB participants of European ancestry with WES.

Gene	UKB Phenotype	GHS Phenotype	OR (95% CI)	P-Value
PIEZO1	I83.9, Asymptomatic varicose veins of lower extremities	I83.9, Asymptomatic varicose veins of lower extremities	3.8 (2.2,6.4)	1.5×10^{-6}
FAM160B1	T81.3, Disruption of wound	T81.3, Disruption of wound	13.2 (1.5,116.6)	0.020

Gene	**UKB Phenotype**	**GHS Phenotype**	**Beta (95% CI)**	**P-Value**
COL6A1	Corneal resistance factor mean	NA[1]	-	-
COL6A1	Corneal hysteresis mean	NA[1]	-	-
GMPR	Mean corpuscular haemoglobin	Mean corpuscular volume	0.18 (0.09,0.27)	8.0×10^{-5}
IQGAP2	Mean platelet thrombocyte volume	Mean platelet volume	0.35 (0.21,0.49)	7.8×10^{-7}
MEPE	Heel bone mineral density	Femoral neck BMD T-score	-0.19 (-0.48,0.11)	0.22

Supplementary Table 14 | Replication of novel LOF burden associations in DiscovEHR. Meta-analysis results using fixed effects models in Plink1.9 for DiscovEHR study participants for corresponding traits for novel UK Biobank LOF gene burden results included in the main text.

[1]No comparable phenotype was available for testing in DiscovEHR.

Variant	Study	Phenotype	RR|RA|AA	Beta (95% CI)	P
rs753138805	UKB 500k imputed	Heel bone mineral density T-score	408698|375|0	-0.41 (-0.50,-0.32)	$2.0x10^{-18}$
rs753138805	HUNT	BMD	19401|303|1	-0.53 (-0.65,-0.41)	$2.1x10^{-18}$
**Variant**	**Study**	**Phenotype**	**RR|RA|AA**	**OR (95% CI)**	**P**
rs753138805	UKB 500k imputed	M81, Osteoporosis without current pathological fracture	Ctrl:452235|406|0 Case:3478|6|0	1.9 (0.9,4.2)	0.10
rs753138805	HUNT	Any Fracture	Ctrl:44936|543|1 Case:23753|402|0	1.4 (1.2,1.5)	$1.6x10^{-5}$
rs753138805	HUNT	Fracture of ankle and foot	Ctrl:44936|543|1 Case:5368|110|0	1.8 (1.4,2.4)	$3.3x10^{-6}$
rs753138805	HUNT	Fracture of hand or wrist	Ctrl:44936|543|1 Case:5761|102|0	1.5 (1.2,1.9)	$7.8x10^{-4}$
rs753138805	HUNT	Fracture of upper limb	Ctrl:44936|543|1 Case:10927|201|0	1.5 (1.3,1.8)	$1.2x10^{-5}$
rs753138805	HUNT	Fracture of unspecified bones	Ctrl:44936|543|1 Case:8480|147|0	1.5 (1.2,1.8)	$3.4x10^{-4}$
rs753138805	HUNT	Fracture of lower limb	Ctrl:44936|543|1 Case:7732|133|0	1.4 (1.1,1.8)	$1.7x10^{-3}$
rs753138805	HUNT	Fracture of pelvis	Ctrl:44936|543|1 Case:799|17|0	1.9 (1.0,3.5)	0.043
rs753138805	HUNT	Fracture of vertebral column without mention of spinal cord injury	Ctrl:44936|543|1 Case:1990|33|0	1.4 (0.9,2.0)	0.14
rs753138805	HUNT	Torus fracture	Ctrl:43203|519|1 Case:805|13|0	1.4 (0.7,2.7)	0.30
rs753138805	HUNT	Fracture of ribs	Ctrl:44936|543|1 Case:1635|25|0	1.2 (0.9,1.9)	0.38
rs753138805	HUNT	Skull and face fracture and other intercranial injury	Ctrl:65020|889|1 Case:1922|29|0	1.1 (0.8,1.7)	0.58

Supplementary Table 15 | Results of *MEPE* rs753138805 associations in UKB 500k imputed and Nord-Trøndelag Health Study (HUNT). The first of two *MEPE* LOFs with the most significant single variant associations with BMD, rs753138805 (p-value = $1.4x10^{-3}$), encodes a four base-pair deletion that leads to an early truncation. We tested this variant (Imputation R^2 = 0.71) for association with BMD and osteoporosis in all European-descent UKB participants with imputed sequence and phenotype of interest available. Replication of the rs753138805 association with BMD and extension to fractures was completed in HUNT (Imputation R^2 = 0.99). rs778732516, which encodes a single base-pair deletion in *MEPE* (BMD p-value =$6.2x10^{-5}$) was not present in the UKB imputed sequence nor HUNT. Effect size

for BMD measures are in standard deviations from the mean. Effect size for fractures and osteoporosis are odds ratios.

Gene	# Non-carriers	Mean Non-carrier Age (SE)	# Carriers	Mean Carrier Age (SE)	P
ASXL1	46809	64.8 (0.072)	102	70.3 (1.1)	$2x10^{-16}$
CHEK2	46599	64.8 (0.072)	312	64.6 (0.91)	0.61
COL4A4	46824	64.8 (0.072)	87	64.2 (1.7)	0.50
COL6A1	46882	64.8 (0.071)	29	64.5 (2.5)	0.84
FAM160B1	46900	64.8 (0.071)	11	69.8 (3.5)	0.02
GMPR	46718	64.8 (0.072)	193	63.9 (1.2)	0.15
GP1BA	46811	64.8 (0.072)	100	63.6 (1.6)	0.14
HBB	46906	64.8 (0.071)	5	57.8 (7.8)	0.15
IL33	46385	64.8 (0.072)	526	64.6 (0.68)	0.51
IQGAP2	46741	64.8 (0.072)	170	64.8 (1.2)	0.93
KALRN	46677	64.8 (0.072)	234	64.4 (0.97)	0.48
KLF1	46881	64.8 (0.071)	30	64.3 (2.5)	0.72
MEPE	46746	64.8 (0.072)	165	65 (1.2)	0.70
MLH1	46889	64.8 (0.071)	22	62.2 (3.9)	0.21
PIEZO1	46746	64.8 (0.072)	165	65.1 (1.1)	0.61
PKD1	46889	64.8 (0.071)	22	66.7 (3.1)	0.24
TTN	46279	64.8 (0.072)	632	65.2 (0.62)	0.22
TUBB1	46878	64.8 (0.071)	33	65.3 (2.5)	0.67

Supplementary Table 16 | Average age of carriers vs non-carriers of LOF variants for genes with a burden association $P < 10^{-7}$. Significance assessed by two tailed two-sample t-test in R.

Gene	Phenotype	Exome 50k P	Imputed 50k P	Imputed 500k P
ASXL1	Platelet distribution width	$1.8x10^{-9}$	NA[1]	NA[1]
ASXL1	Red blood cell erythrocyte distribution width	$6.7x10^{-9}$	NA[1]	NA[1]
COL4A4	MCR noCKD	$8.2x10^{-10}$	0.84	0.73
CHEK2	Platelet crit	$9.8x10^{-8}$	0.054	$1.4x10^{-3}$
COL6A1	Corneal resistance factor mean	$4.7x10^{-10}$	NA[1]	NA[1]
COL6A1	Corneal hysteresis mean	$2.9x10^{-8}$	NA[1]	NA[1]
FAM160B1	T81.3, Disruption of wound	$9.4x10^{-8}$	NA[1]	NA[1]
GMPR	Mean corpuscular haemoglobin	$3.8x10^{-8}$	$2.6x10^{-5}$	$9.4x10^{-32}$
GP1BA	Mean platelet thrombocyte volume	$7.7x10^{-8}$	NA[1]	NA[1]
HBB	Red blood cell erythrocyte distribution width	$7.4x10^{-8}$	0.70	0.20
HBB	Red blood cell erythrocyte count	$1.7x10^{9}$	0.23	0.30
IL33	Eosinophil percentage	$2.4x10^{-11}$	$2.7x10^{-11}$	$1.2x10^{-85}$
IL33	Eosinophil count	$1.3x10^{-10}$	$6.8x10^{-10}$	$7.8x10^{-79}$
IQGAP2	Mean platelet thrombocyte volume	$9.2x10^{-22}$	0.11	$1.1x10^{-9}$
KALRN	Mean platelet thrombocyte volume	$5.8x10^{-24}$	$1.7x10^{-20}$	$2.0x10^{-120}$
KLF1	Red blood cell erythrocyte distribution width	$2.0x10^{-13}$	0.43	0.76
KLF1	Mean corpuscular haemoglobin	$1.2x10^{-15}$	0.79	0.038
KLF1	Mean corpuscular volume	$5.9x10^{-14}$	0.66	0.026
MEPE	Heel BMD T-score	$1.9x10^{-8}$	$3.2x10^{-3}$	$2.2x10^{-14}$
MLH1	Z85.0, Personal history of malignant neoplasm of digestive organs	$8.2x10^{-11}$	NA[2]	0.80
PIEZO1	I83.9, Asymptomatic varicose veins of lower extremities	$3.2x10^{-8}$	0.083	0.28
PKD1	N18, Chronic kidney disease	$6.4x10^{-10}$	NA[1]	NA[1]
TTN	I42, Cardiomyopathy	$6.7x10^{-9}$	0.016	0.054
TUBB1	Platelet distribution width	$1.2x10^{-22}$	$5.3x10^{-3}$	$4.0x10^{-23}$
TUBB1	Mean platelet thrombocyte volume	$3.6x10^{-8}$	0.028	$1.4x10^{-10}$
TUBB1	Platelet count	$2.1x10^{-9}$	0.029	$1.2x10^{-8}$

Supplementary Table 23 | Comparison of LOF burden associations in 50k exome vs 50k imputed vs 500k imputed.

[1]No LOFs were available for testing in imputed data.

[2]Did not reach MAC >3 threshold for inclusion in analysis.

n, number of carriers	Observed number of genes with ≥n heterozygous LOF variant carriers in 50k	Predicted number of genes with ≥n heterozygous LOF variant carriers in 50k	Predicted number of genes with ≥n heterozygous LOF variant carriers in 500k
1	17,718	17,678	18,273
5	15,253	15,064	18,030
10	12,598	12,308	17,731
25	7,764	7,452	16,779
50	4,555	4,301	15,202
100	2,443	2,287	12,489

Supplementary Table 25 | Predicted number of genes with heterozygous LOF carriers in larger WES sample sizes from existing 50k WES data. The number of autosomal genes with at least 1, 5, 10, etc. heterozygous LOF carriers passing Goldilocks QC and genotype missingness<10%, and HWE p-value>10^{-15} in 46,911 UKB participants of European ancestry with WES, and predicted number of genes with N heterozygous LOF carriers in ~50k (46,911) and 500k individuals.

LOF filtering and transcript definition	UKB WES European, n=46,911		gnomAD NFE, n=56,885	
	#LOFs	#Genes	#LOFs	#Genes
RGC LOF Definition	202,565	17,718	260,431	17,946
LOFTEE LOF Definition	172,564	17,323	223,492	17,620

Supplementary Table 26 | LOF variants in UKB and gnomAD using RGC annotation pipeline. Number of autosomal predicted loss of function (LOF) variants MAF <1% in targeted and non-targeted WES and number of genes with at least one heterozygous LOF in UKB participants of European ancestry, n=46,911 and gnomAD Non-Finnish-Europeans, n=56,885. MAF was estimated and applied within UKB and gnomAD samples, respectively.

Characteristics	Definition
Body mass index	Field ID: 21001, Instance 0
Number of current/past smokers	Field ID: 20116, Status ½, Instance 0
Townsend Deprivation Index	Field ID: 189, Instance 0
Inpatient ICD10 codes per patient	Field IDs: 41142 (primary diagnosis), 41078 (secondary diagnosis) 1 or more ICD 3D primary or secondary diagnoses in HES data
Percentage of patients with >=1 ICD10 diagnoses	Field IDs: 41142 (primary diagnosis), 41078 (secondary diagnosis) Percentage of patients with 1 or more primary or secondary diagnoses (ICD 3D) in HES data
**Cardiometabolic phenotypes**	**ICD10 codes, self-reported and doctor-diagnosed code**
Coronary Disease	I20 OR I21 I22 I23 OR I24 OR I25 OR 42000_0__0_Date_of_first_myocardial_infarction_CASE (!=NA) OR Z951 OR Z955
Heart Failure	I50
Type 2 Diabetes	E11
**Respiratory and immunological phenotypes**	
Asthma	J45 OR J46 OR Self_reported_asthma (1111) OR Doctor_diagnosed_asthma
COPD	J41 OR J42 OR J43 OR J44
Rheumatoid Arthritis	M05 OR M06 OR Self-reported_Rheumatoid_Arthritis (1464)
Inflammatory Bowel Disease	K50 OR K51 OR Self-reported_Ulceritive_Colitis (1463)
**Neurodegenerative phenotypes**	
Alzheimer's Disease	G30
Parkinson's Disease	G20
Multiple Sclerosis	G35
Myasthenia Gravis	G70
**Oncology phenotypes**	
Breast cancer	HES(C50) OR Self-Reported_Breast_Cancer (1002) OR Cancer Registry (C50)
Ovarian cancer	HES(C56) OR Self-Reported_Ovarian_Cancer (1039) OR Cancer Registry (C56)
Prostate cancer	HES(C61) OR Self-Reported_Prostate_Cancer (1026) OR Cancer Registry (C61)
Pancreatic cancer	HES(C25) OR Self-Reported_Pancreatic_Cancer (1044) OR Cancer Registry (C50)
Melanoma	HES(C43) OR Self-Reported_Melanoma (1049) OR Cancer Registry (C43)

Lung cancer	HES(C34) OR Self-Reported_Lung_Cancer (1001) OR Cancer Registry (C34)
Colorectal cancer	HES(C18) OR Self-Reported_Colorectal_Cancer (1049) OR Cancer Registry (C18)
Cutaneous squamous cell carcinoma (CSCC)	HES(C44) OR Self-Reported_Squamous_Cell_Carcinoma (1062) OR Cancer Registry (C44)
**Enhanced measures**	**Field ID**
Pulse rate	4194 Instance 0
Visual Acuity Measured	5187 Instance 0
IOP measured (left)	5262 Instance 0
Autorefraction	5159 Instance 0
Retinal OCT	6072 Instance 0
ECG at rest	22334
Cognitive Function	20242, Fluid intelligence completion status
Digestive Health	21066, Born by caesarian section
Physical Activity Measurement	90112, Fraction acceleration <= 25 milli-gravities

Supplementary Table 29 | Definition of UK Biobank phenotypes. Disease phenotype cases were defined using >=1 primary or >=2 secondary diagnoses from HES data.

Phenotype	ICD9	ICD10
Fracture of lower limb	820-823;825;827;905;V54	S72;S82;S92;T02;T12
Fracture of ankle and foot	824-826	S82;S92
Fracture of pelvis	808	S32
Fracture of upper limb	810-813;818-819;905;V54	S22;S42;S52;T02;T10;T92
Fracture of hand or wrist	814-817	S52;S62
Fracture of vertebral column without mention of spinal cord injury	805-806;905;V54	M49;S12;S22;S32;T02;T91
Fracture of ribs	807	S22
Fracture of unspecified bones	807;809;828-829;905;V54;V66-67	S22;S32;T02;Z09;Z54
Skull and face fracture and other intercranial injury	800-804;854;905;907;V15	S02;S06;T02;T06;T90
Torus fracture	823	

Supplementary Table 31 | Definition of HUNT fracture phenotypes. Fracture phenotype cases were defined using ICD9 and ICD10 codes.

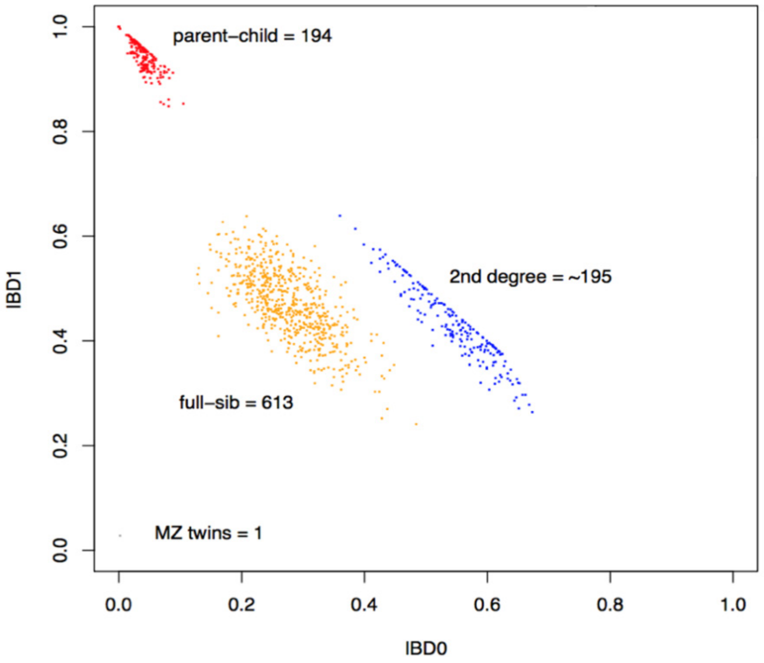

Supplementary Figure 1 | Distribution of IBD sharing for pairs of individuals in UKB 50k WES. Estimated proportion of WES genotypes with no alleles identical by descent (IBD) vs. 1 allele IBD amongst all pairs of UKB 50k exome participants. Note that 2nd relationship pairs (blue) are included only if they are part of a relationship network containing a parent-child (red) full-sib (orange) or monozygotic twin (grey) relationship pair. We calculated IBD proportions in PLINK, restricting the analysis to common single nucleotide polymorphisms with minor allele frequency >10%, genotype missingness <5%, and a Hardy-Weinberg Equilibrium p-value > 0.00001.

a b

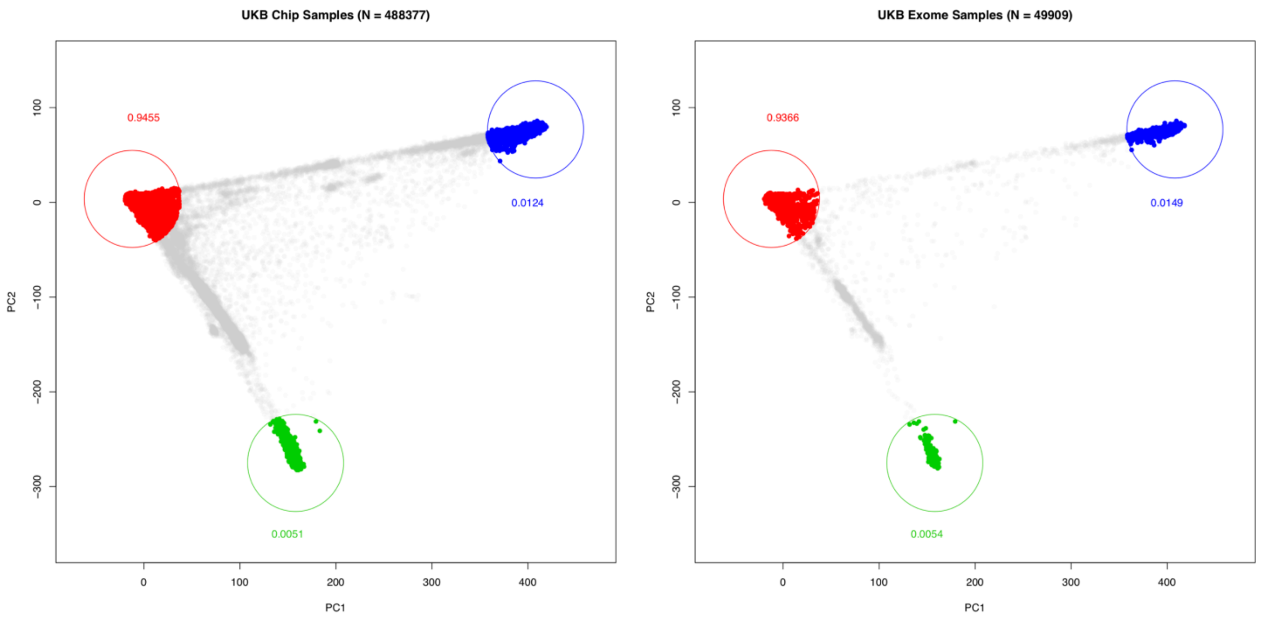

<u>Supplementary Figure 2 | Continental ancestry in UK Biobank 500k and 50k.</u> Principal component 1 and 2 for **a,** n=488,377 and **b,** individuals with WES available from the UK Biobank Data Showcase. Three pre-defined regions of a plot of represent African (blue), East Asian (green), and European (red) ancestry.

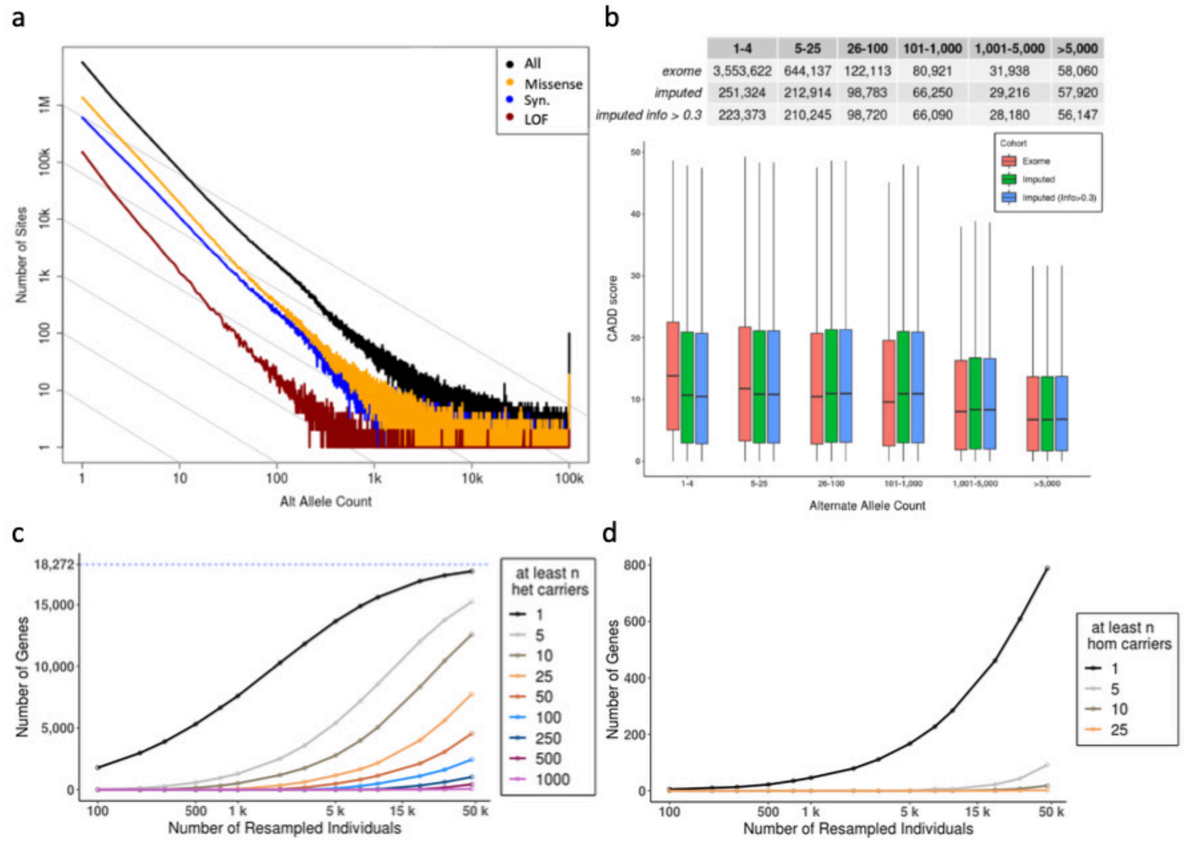

Supplementary Figure 3 | Summary statistics for variation in WES and sequence imputed from array. **a,** Observed site frequency spectrum for all autosomal variants and by functional prediction in 49,960 UKB participants. **b,** Distribution of CADD scores for variant allele counts in regions consistently covered by WES (90% of individuals with >20X depth) in WES and sequence imputed from array in 49,797 UKB participants with WES and imputed sequence. Box plot elements include; center line, median; box limits, upper and lower quartiles; upper whisker 1.5x interquartile range; lower whisker, 0. **c,** The number of autosomal genes with at least 1, 5, 10, etc. heterozygous and **d,** homozygous LOF variant carriers increases with sample size. All exome variants passed GL (Goldilocks) QC (see Supplementary Information for GL QC filtering definition), genotype missingness<10%, and HWE p-value>10^{-15}. 46,911 UKB participants of European ancestry with WES were down sampled at random to the number of individuals specified on the horizontal axis. The number of genes containing at least the indicated count of LOFs MAF<1% carriers as in the legend are plotted on the vertical axis. The maximum number of autosomal genes is 18,272 in this analysis (See Supplementary Information for gene model).

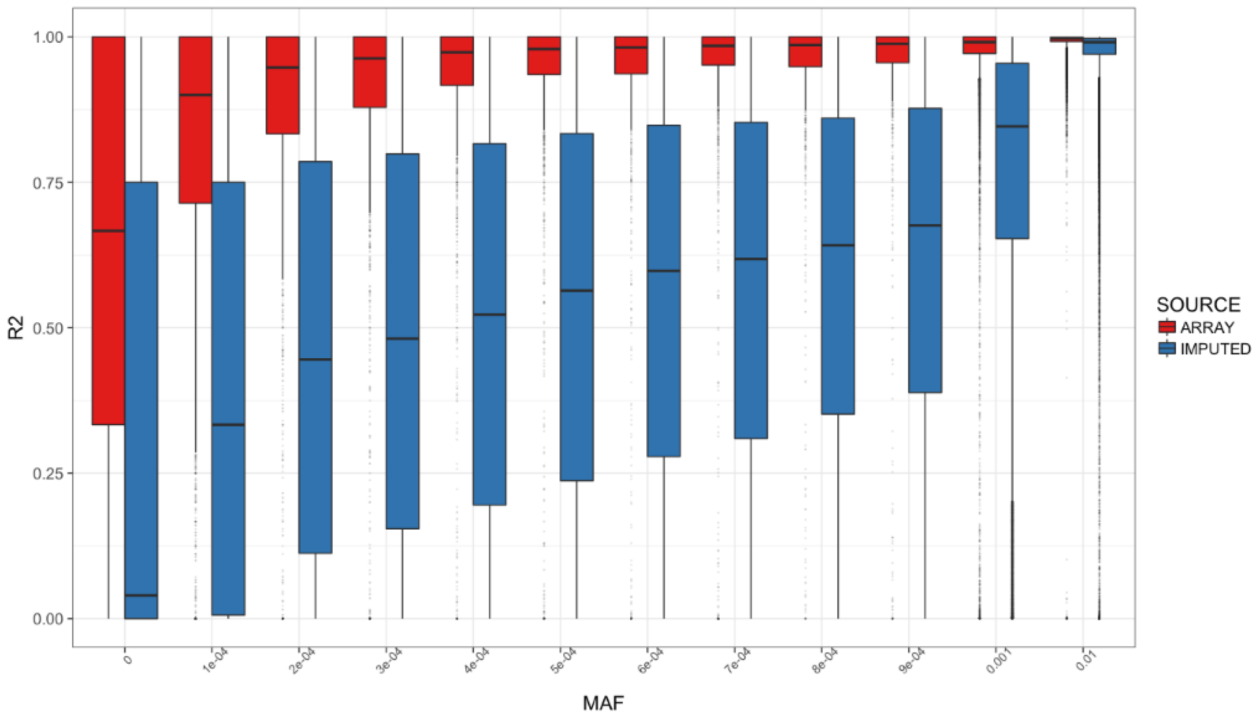

<u>Supplementary Figure 4 | R^2 Concordance between WES-Array and WES-Imputed datasets</u>. R-squared correlation coefficients as metrics of concordance between variants in WES and array genotypes (red) and sequence imputed from array (imputation score >0.3) (blue), calculated per variant and binned by minor allele frequency (MAF) in WES. All MAF bins are left inclusive and right exclusive, except for the 1%-50% bin which includes both boundaries. The median R-squared correlation coefficient is represented by the horizontal black bars; interquartile range represented by the colored boxes; 5-95th quartiles represented by the black box-whiskers and observations outside this range represented by black dots. In total, n=46,806 individuals were used for both the array-WES comparison and the imputed-WES comparison. N=97,358 variants were represented in the array-WES comparison, and n=982,234 variants were represented in the imputed-WES comparison.

a b

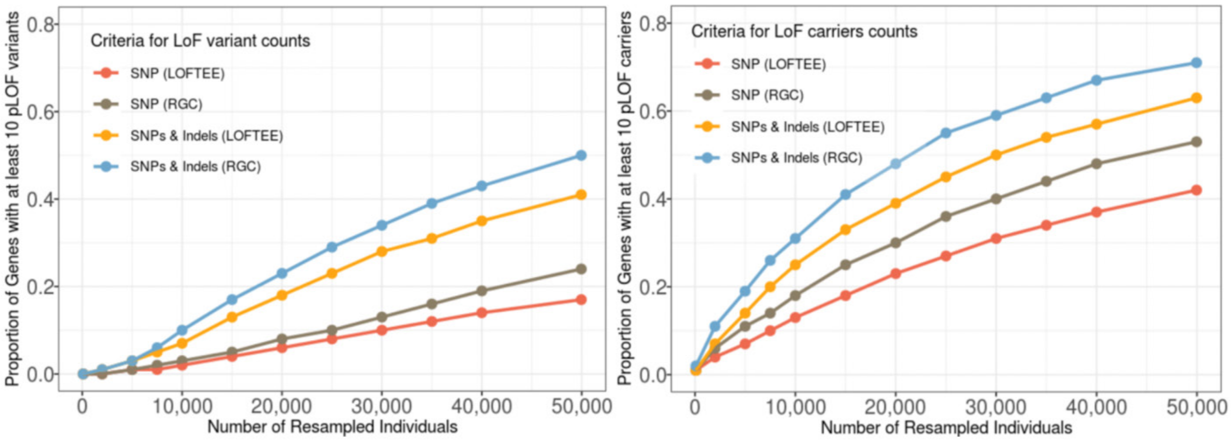

Supplementary Figure 5 | Accumulation of genes with at least 10 LOF variants and carriers as a function of sample size. 49,960 UKB participants with WES were down sampled at random to the number of individuals specified on the horizontal axis. The number of genes containing **a,** at least 10 LOF *variants* (each LOF variant counted once, even if observed in multiple individuals) and **b,** at least 10 LOF *variant carriers* (each LOF genotype counted once) according to criteria indicated in the legend are plotted on the vertical axis, i.e., RGC or LOFTEE LOF definition, SNPs only or SNPs & Indels. All LOFs passed GL quality control criteria and had MAF < 1%. Note that the number of *variant carriers* is nearly always greater than the number of *variants*.

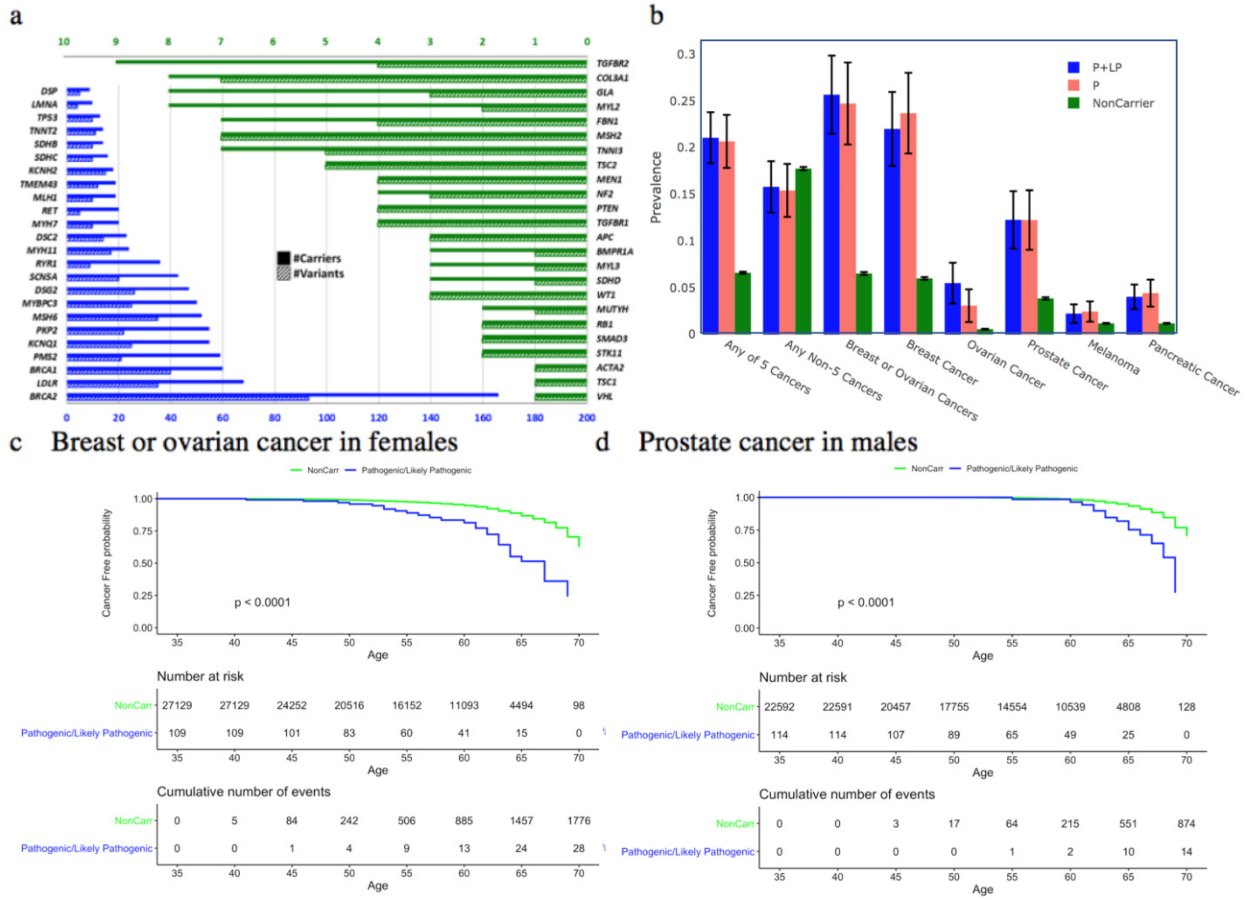

Supplementary Figure 6 | Summary of observed actionable ACMG59 variants, and pathogenicity of *BRCA1/2* variants. a, Counts of variants and carriers in 47 ACMG59 genes with pathogenic (P) or likely pathogenic (LP) variants. **b,** Prevalence and standard error of cancers in carriers of P, P or LP, and no P or LP variants in *BRCA1* or *BRCA2*. Five major cancers related to *BRCA1/2* risk include breast in females, ovarian, prostate, melanoma, and pancreatic cancers. Cases are aggregated from Cancer Registry, HES, and Self Report. See Supplementary Table 11 for LP,P, and non-carrier case and control counts. Kaplan Meier survival analysis curves of the **c,** cumulative proportion of female participants free of breast and ovarian cancer, and **d,** male participants free of prostate cancer with P or LP variants in *BRCA1/2* compared to non-carriers by age at interview.

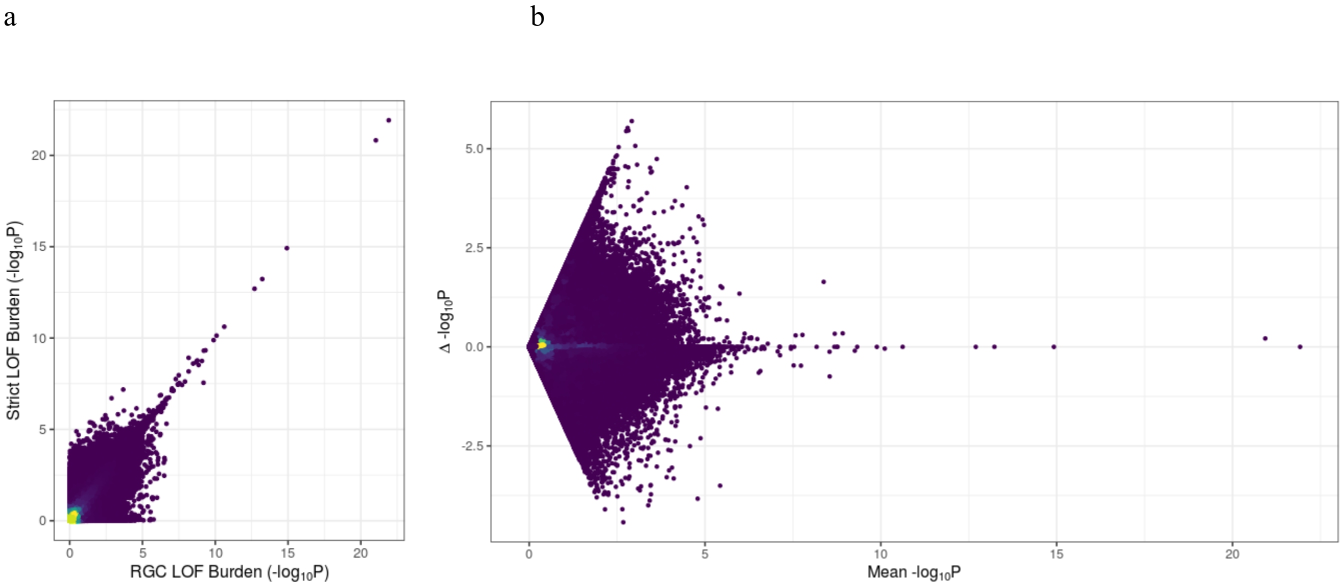

Supplementary Figure 7 | Comparison of association results using RGC and LOFTEE LOF definitions. For the 19,013,714 results that were generated using RGC and LOFTEE LOF definitions a, scatterplot of -Log10 P-value of RGC and LOFTEE LOF definitions across all computed associations. b, Bland-Altman plot of difference in -log10P between RGC and LOFTEE LOF definitions vs mean -log10P across all computed associations.

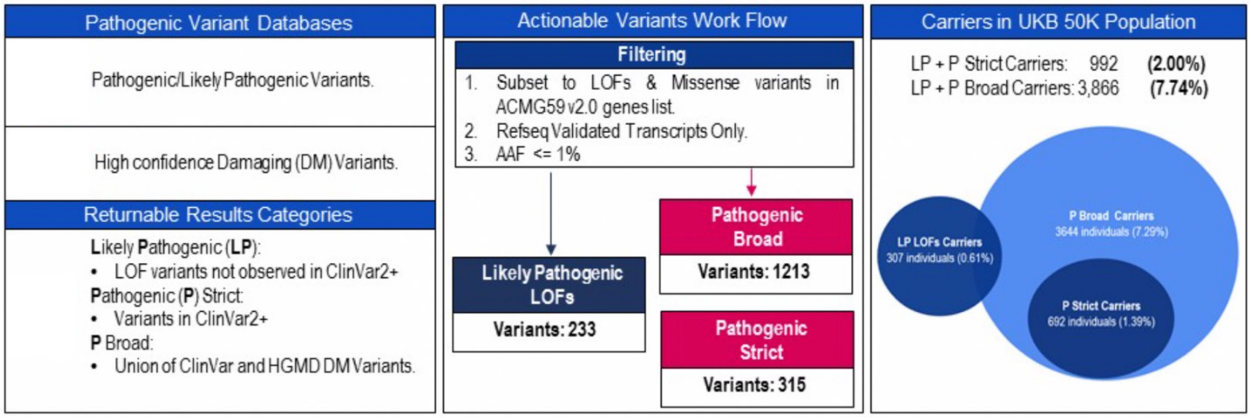

Supplementary Figure 8 | Actionable Variants Work Flow. Visualization of characterization of medically actionable variants.
