# Supplementary material for: Exome sequencing and characterization of 49,960 individuals in the UK Biobank
Source: Nature. 2020 Oct 21;586(7831):749–56. doi: 10.1038/s41586-020-2853-0 (PMC7759458; doi:10.1038/s41586-020-2853-0)
Supplement: Supplementary file 2 — Reporting Summary [file 41586_2020_2853_MOESM2_ESM.pdf]

nature research

Corresponding author(s): Cristopher V. Van Hout, Aris Baras, Laura Yerges-Armstrong, Robert Scott

Last updated by author(s): May 7, 2020

Reporting Summary

Nature Research wishes to improve the reproducibility of the work that we publish. This form provides structure for consistency and transparency in reporting. For further information on Nature Research policies, see Authors & Referees and the Editorial Policy Checklist.

Statistics

For all statistical analyses, confirm that the following items are present in the figure legend, table legend, main text, or Methods section.

n/a	Confirmed	
☐	☒	The exact sample size (*n*) for each experimental group/condition, given as a discrete number and unit of measurement
☐	☒	A statement on whether measurements were taken from distinct samples or whether the same sample was measured repeatedly
☐	☒	The statistical test(s) used AND whether they are one- or two-sided<br>*Only common tests should be described solely by name; describe more complex techniques in the Methods section.*
☐	☒	A description of all covariates tested
☐	☒	A description of any assumptions or corrections, such as tests of normality and adjustment for multiple comparisons
☐	☒	A full description of the statistical parameters including central tendency (e.g. means) or other basic estimates (e.g. regression coefficient) AND variation (e.g. standard deviation) or associated estimates of uncertainty (e.g. confidence intervals)
☐	☒	For null hypothesis testing, the test statistic (e.g. *F*, *t*, *r*) with confidence intervals, effect sizes, degrees of freedom and *P* value noted<br>*Give P values as exact values whenever suitable.*
☒	☐	For Bayesian analysis, information on the choice of priors and Markov chain Monte Carlo settings
☒	☐	For hierarchical and complex designs, identification of the appropriate level for tests and full reporting of outcomes
☐	☒	Estimates of effect sizes (e.g. Cohen's *d*, Pearson's *r*), indicating how they were calculated

Our web collection on statistics for biologists contains articles on many of the points above.

Software and code

Policy information about availability of computer code

Data collection

Data transfer from sequencing machine to DNAnexus
-Upload Agent v1.5.30 https://wiki.dnanexus.com/Downloads#Upload-Agent

Single-sample processing, all in DNAnexus
-Conversion of sequencing data in BCL format to FASTQ format and the assignments of paired-end sequence reads to samples based on 10-base barcodes; bcl2fastq v2.19.0 https://support.illumina.com/sequencing/sequencing_software/bcl2fastq-conversion-software.html
-Read alignment; bwa 0.7.17 http://bio-bwa.sourceforge.net
-Duplicate marking, stats gathering; picard v1.141 https://broadinstitute.github.io/picard/
-SAM/BAM/CRAM file generation and manipulation; samtools v1.7 http://www.htslib.org
-Variant calling; WeCall v1.1.2 https://github.com/Genomicsplc/wecall
-Sequence Quality Control; FastQC 0.11.8 http://www.bioinformatics/babraham.ac.uk/projects/fastqc/
-VCF file manipulation and index generation; bcftools v1.7 http://www.htslib.org, bgzip/tabix v1.7 http://www.htslib.org
-Multi-threaded file compression and decompression; pigz v2.3.4 https://zlib.net/pigz/
Generation of “freeze” data
-Joint genotyping to generate project-level VCF (pVCF) files; GLnexus v0.4.0 https://github.com/dnanexus-rnd/GLnexus
-Generation of variant representations in PLINK format; PLINK v1.90b3.37 https://www.cog-genomics.org/plink2/
-Ancestry predictions, IBD (Identity-by-descent) estimate, and pedigree reconstruction; PLINK v1.90b3.37 https://www.cog-genomics.org/plink2/, PRIMUS https://primus.gs.washington.edu/primusweb/

Data analysis

-Single variant and burden tests for quantitative traits; BOLT-LMM_v2.3.2 https://data.broadinstitute.org/alkesgroup/BOLT-LMM/
-Single variant and burden tests for binary outcomes; SAIGE_v0.29.1 https://github.com/weizhouUMICH/SAIGE
-GHS quantitative results; PLINK v1.90b3.38 64-bit (7 Jun 2016) https://www.cog-genomics.org/plink/1.9/
-Various, including GHS meta analyses; PLINK v1.90b3.45 64-bit (13 Jan 2017) https://www.cog-genomics.org/plink/1.9/
-Imputed sequence conversion; PLINK v2.00a2LM AVX2 Intel (31 Mar 2018) https://www.cog-genomics.org/plink/2.0/

-Various; R v3.4.3 https://cran.r-project.org

For manuscripts utilizing custom algorithms or software that are central to the research but not yet described in published literature, software must be made available to editors/reviewers. We strongly encourage code deposition in a community repository (e.g. GitHub). See the Nature Research guidelines for submitting code & software for further information.

Data

Policy information about availability of data

All manuscripts must include a data availability statement. This statement should provide the following information, where applicable:

- Accession codes, unique identifiers, or web links for publicly available datasets
- A list of figures that have associated raw data
- A description of any restrictions on data availability

The UK Biobank related datasets generated during and/or analyzed in the current study are available to any bona fide researcher from the UK Biobank Showcase, https://biobank.ctsu.ox.ac.uk/showcase/ UK Biobank data for this report were obtained under data access application 26041.

Field-specific reporting

Please select the one below that is the best fit for your research. If you are not sure, read the appropriate sections before making your selection.

☒ Life sciences ☐ Behavioural & social sciences ☐ Ecological, evolutionary & environmental sciences

For a reference copy of the document with all sections, see nature.com/documents/nr-reporting-summary-flat.pdf

Life sciences study design

All studies must disclose on these points even when the disclosure is negative.

Sample size	Sample sizes were all those available in UK Biobank, or subsetted to those with available exome sequencing, as described in the text. Further subsetting was applied based on the specific use case, for example, results include individuals of European ancestry defined by principal components and clustering, and concordance analysis includes individuals with available exome and imputed sequence, as described in the text. Sample sizes for replication studies included all individuals available from their respective samples as detailed in the text. No power calculations were performed or required in advance.
Data exclusions	This analysis excludes whole exome sequencing variants that do not pass 'Goldilocks' QC, as detailed in Supplemental Methods. Results in UK Biobank excluded individuals of non-European ancestry as defined by principal components and clustering as detailed in the text and Supplemental Methods.
Replication	We replicated UK Biobank gene burden association results in the Regeneron-Geisinger DiscovEHR, and results related to MEPE in the HUNT study, as described in the text and supplemental material.
Randomization	This study is observational. No process of randomization of experimental groups was performed or applicable to this study.
Blinding	This study is is observational, using coded de-identified data. No process of blinding to group allocation was performed or applicable to this study.

Reporting for specific materials, systems and methods

We require information from authors about some types of materials, experimental systems and methods used in many studies. Here, indicate whether each material, system or method listed is relevant to your study. If you are not sure if a list item applies to your research, read the appropriate section before selecting a response.

Materials & experimental systems

n/a	Involved in the study	
☒	☐	Antibodies
☒	☐	Eukaryotic cell lines
☒	☐	Palaeontology
☒	☐	Animals and other organisms
☐	☒	Human research participants
☒	☐	Clinical data

Methods

n/a	Involved in the study	
☒	☐	ChIP-seq
☒	☐	Flow cytometry
☒	☐	MRI-based neuroimaging

Human research participants

Policy information about studies involving human research participants

Population characteristics	UK Biobank is a general population prospective epidemiological study comprising approximately 500,000 individuals, age 40-69

Population characteristics	years of age at recruitment. See Table 1 of the manuscript for clinical and demographic characteristics for the subset of UK Biobank with WES and all of the UKB participants. Additional population characteristics are available to the public at http://www.ukbiobank.ac.uk/
Recruitment	UK Biobank recruited approximately 500,000 individuals 40-69 years of age in 2006 to 2010 by mailers to people in the UK medical system. Informed consent was obtained for all participants.
Ethics oversight	The scientific protocol of the UK Biobank is approved from appropriate external ethics committees in accordance with guidance from relevant bodies. For additional information, see https://www.ukbiobank.ac.uk/wp-content/uploads/2011/05/EGF20082.pdf

Note that full information on the approval of the study protocol must also be provided in the manuscript.
